# Early neuronal accumulation of DNA double strand breaks in Alzheimer’s disease

**DOI:** 10.1186/s40478-019-0723-5

**Published:** 2019-05-17

**Authors:** Niraj M. Shanbhag, Mark D. Evans, Wenjie Mao, Alissa L. Nana, William W. Seeley, Anthony Adame, Robert A. Rissman, Eliezer Masliah, Lennart Mucke

**Affiliations:** 1 Gladstone Institute of Neurological Disease, San Francisco, CA 94158 USA; 2 Memory and Aging Center, Department of Neurology, University of California San Francisco, San Francisco, CA 94158 USA; 3 Department of Pathology, University of California San Francisco, San Francisco, CA 94158 USA; 4 Department of Neurosciences, University of California at San Diego, La Jolla, CA 92093 USA; 5 Present address: Division of Neuroscience, National Institute on Aging, Bethesda, MD 20892 USA

**Keywords:** Alzheimer’s disease, Astrocytes, DNA damage, 53BP1, γH2AX, Neurons

## Abstract

The maintenance of genomic integrity is essential for normal cellular functions. However, it is difficult to maintain over a lifetime in postmitotic cells such as neurons, in which DNA damage increases with age and is exacerbated by multiple neurological disorders, including Alzheimer’s disease (AD). Here we used immunohistochemical staining to detect DNA double strand breaks (DSBs), the most severe form of DNA damage, in *postmortem* brain tissues from patients with mild cognitive impairment (MCI) or AD and from cognitively unimpaired controls. Immunostaining for γH2AX—a post-translational histone modification that is widely used as a marker of DSBs—revealed increased proportions of γH2AX-labeled neurons and astrocytes in the hippocampus and frontal cortex of MCI and AD patients, as compared to age-matched controls. In contrast to the focal pattern associated with DSBs, some neurons and glia in humans and mice showed diffuse pan-nuclear patterns of γH2AX immunoreactivity. In mouse brains and primary neuronal cultures, such pan-nuclear γH2AX labeling could be elicited by increasing neuronal activity. To assess whether pan-nuclear γH2AX represents DSBs, we used a recently developed technology, DNA damage in situ ligation followed by proximity ligation assay, to detect close associations between γH2AX sites and free DSB ends. This assay revealed no evidence of DSBs in neurons or astrocytes with prominent pan-nuclear γH2AX labeling. These findings suggest that focal, but not pan-nuclear, increases in γH2AX immunoreactivity are associated with DSBs in brain tissue and that these distinct patterns of γH2AX formation may have different causes and consequences. We conclude that AD is associated with an accumulation of DSBs in vulnerable neuronal and glial cell populations from early stages onward. Because of the severe adverse effects this type of DNA damage can have on gene expression, chromatin stability and cellular functions, DSBs could be an important causal driver of neurodegeneration and cognitive decline in this disease.

**Electronic supplementary material:**

The online version of this article (10.1186/s40478-019-0723-5) contains supplementary material, which is available to authorized users.

## Introduction

Genomic integrity is necessary for normal gene expression and cellular functions. However, all cells in the body are continually subjected to DNA damage from endogenous and exogenous sources [16, 53, 65]. The cellular DNA repair machinery evolved to reverse this damage. Nonetheless, aging is associated with the accumulation of DNA damage across diverse cell types, tissues and animal species, and much evidence suggests that DNA damage accelerates aging and related impairments [55, 57, 71, 83].

The ability to maintain genomic integrity is probably most important for postmitotic cells that are long lived such as neurons. Unable to divide, and for the most part irreplaceable, the majority of neurons have to rely on their genetic material for the lifetime of an organism. Furthermore, neurons have a highly active metabolism and produce large quantities of free radicals, which can cause oxidative DNA damage [25]. Notably, increased levels of DNA damage in aging brains are associated with the reduced expression of essential genes, including genes involved in neuronal plasticity [62]. Neuronal DNA damage is further exacerbated in many neurodegenerative disorders [6, 42, 65, 92], which may contribute to the extensive changes in gene expression and neuronal loss found in these conditions.

Numerous studies have reported increased evidence for oxidative DNA damage in the brains, cerebrospinal fluid, and peripheral cells of patients with mild cognitive impairment (MCI) or Alzheimer’s disease (AD) [6, 22, 31, 58–60, 115]. This DNA damage has been attributed to increased production of free radicals and to deficits in relevant DNA repair pathways [8, 58]. Most often, oxidative DNA damage appears to consist primarily of DNA base modifications and single strand breaks [12, 27].

Interestingly, much less is known about the association between AD and DNA double strand breaks (DSBs), the most deleterious form of DNA damage. In dividing cells, unrepaired DSBs can lead to cell cycle arrest and cell death [120], while the inaccurate repair of DSBs can lead to mutations, deletions, or chromosomal translocations [20, 110]. Unlike dividing cells, which can utilize sister chromatids to repair DSBs in an error-free manner by homologous recombination, postmitotic neurons must resort to more error-prone mechanisms of DSB repair such as nonhomologous end joining (NHEJ) [39, 51]. Thus, DSBs could be particularly detrimental to neuronal function and survival, and thereby promote the development of age-related cognitive decline or disease [65].

The response to DSB formation involves the rapid post-translational modification and recruitment of multiple proteins to broken DNA ends and the surrounding chromatin. One of the earliest events in this process is the phosphorylation of the histone variant H2AX at serine 139, which occurs at multiple sites for up to megabases of chromatin surrounding the break site [5, 35, 91, 99]. Nuclear foci containing high concentrations of phospho-S139-H2AX, termed γH2AX, can be visualized by immunohistochemistry and are widely used as putative evidence for the presence of DSBs [54, 91]. Many DSB response proteins, including 53BP1, are also recruited to and retained at sites of DSBs at high concentrations and can be visualized with similar methods.

Current reports of DSBs in the brains of patients with AD are conflicting. One population-based study demonstrated γH2AX immunoreactivity in neurons and glia of patients with low levels of AD pathology and suggested a trend toward decreasing γH2AX levels as AD pathology increased [100]. The same group showed that γH2AX immunoreactivity could be detected in brain cells of aged people with little or no AD pathology, and that the number of γH2AX-positive neurons inversely correlated with bedside cognitive test scores [101]. In contrast, another group found evidence for γH2AX immunoreactivity in astrocytes, but not neurons, in brains from patients with advanced AD pathology [76].

Notably, these studies used immunoperoxidase staining to detect γH2AX and showed diffuse, pan-nuclear staining patterns, as opposed to the focal pattern seen at genuine DSBs [54, 90]. More recently, Mano and colleagues complemented γH2AX immunoperoxidase staining with the neutral comet assay [79, 80, 102] to demonstrate increased levels of DSBs in a small number of AD patients [68]. However, the neutral comet assay does not allow for a determination of the cell types that harbor DSBs, can be confounded by topological changes in DNA induced by experimental conditions [3], and relies solely on the presence of broken DNA strands, some of which might result from *postmortem* tissue degradation.

In this study, we used the well-established neutral comet assay, as well as optimized immunohistochemical approaches, novel technologies and high-resolution microscopy on *postmortem* brain tissues from two independent human cohorts to determine whether AD and MCI, a frequent harbinger of AD [46, 67], are associated with an increase in neuronal DSBs. We used mouse and cell culture models to validate our DSB detection methods and to differentiate cellular staining patterns that represent DSBs from those caused by other processes that could confound the interpretation of results obtained by widely used methods.

## Materials and methods

### Human *postmortem* tissues and neuropathological diagnosis

Cohort 1 consisted of 13 cases (Additional file 1: Table S1). Tissues were obtained from the University of California, San Francisco (UCSF) Neurodegenerative Diseases Brain Bank. Before their death, subjects had been studied neurologically and psychometrically at the UCSF Memory and Aging Center. Authorization for autopsy was provided by all subjects or their next-of-kin in accordance with the Declaration of Helsinki, and all procedures were approved by the UCSF Committee on Human Research. At autopsy, the cerebrum was cut fresh into 1 cm thick coronal slices and fixed for 48–72 h in buffered 10% formalin. Neuropathological diagnoses were made in accordance with consensus diagnostic criteria [33, 70] using histological and immunohistochemical methods as described [108]. Cases were selected based on neuropathological diagnosis. Blocks of the inferior frontal gyrus, pars orbitalis were dissected and placed into PBS with 0.02% sodium azide for storage.

Cohort 2 consisted of 23 cases (Additional file 1: Table S2). Before their death, subjects had been examined neurologically and psychometrically at the Shiley-Marcos Alzheimer’s Disease Research Center/University of California at San Diego (ADRC/UCSD). Authorization for autopsy was provided by all subjects or their next-of-kin in accordance with the Declaration of Helsinki, and all procedures were approved by the UCSD Institutional Review Board. At autopsy, brains were processed and evaluated as described [87]. They were divided sagittally. The left hemibrain was fixed in buffered 10% formalin or 4% paraformaldehyde (PFA) for 2 weeks for histopathological analysis; the right hemibrain was frozen at − 70 °C for DNA damage in situ ligation followed by proximity ligation assay (DI-PLA). Paraffin sections of formalin-fixed neocortical, limbic and other subcortical tissues were stained with hematoxylin and eosin (H&E), or with antibodies against amyloid-β (Aβ) or phosphorylated tau (Additional file 1: Table S3), followed by detection of primary antibodies with species-matched secondary antibodies (Agilent/Dako), development with the immunoperoxidase method, and visualization by light microscopy as described [87]. Neuropathological analysis included counting of amyloid plaques and neurofibrillary tangles (per 0.1 mm^2^), and Braak staging [33, 87].

### Double-immunolabeling and laser scanning confocal microscopy of human brain sections

For Cohort 1, double-immunolabeling studies were performed on free-floating 30-μm microtome sections from the orbitofrontal cortex. Briefly, sections were washed in Tris-buffered saline (TBS), permeabilized in TBS containing 0.5% Triton X-100 (Sigma-Aldrich) at 4 °C overnight, and incubated for 15 min at 110 °C in citrate buffer (pH 6.0) for antigen retrieval. They were then incubated in 3% H_2_O_2_ and 10% methanol for 15 min, 0.1% Sudan Black (Sigma-Aldrich) in 70% ethanol for 15 min, and 10% goat serum in TBS containing 0.1% Tween-20 (TBST) for 1 h. Anti-γH2AX and anti-NeuN antibodies (Additional file 1: Table S3) in TBST containing 3% goat serum were applied first at 4 °C overnight and then at room temperature for 2 h. Primary antibodies were detected with Alexa Fluor secondary antibodies (Thermo Fisher Scientific) at 1:500 dilution. All sections were processed simultaneously under the same conditions, except that for some sections, TO-PRO-3 (Thermo Fisher Scientific) was used to stain nuclei. Sections were mounted onto glass slides and covered with ProLong Diamond Antifade Mountant (Thermo Fisher Scientific).

Confocal images were acquired from cortical layer II/III with an LSM 880 laser-scanning confocal microscope (Zeiss, Germany) and a 20x objective lens (NA 0.8). Z-stack images were taken with sequential acquisition settings at pixel size of 0.41 μm. ImageJ software [93] was used to analyze γH2AX foci. First, the NeuN channel was used to select 50 neurons from a single image per case. In these cells, the γH2AX channel was then used to calculate the number of γH2AX foci per nucleus through manual counting. The proportion of neurons with one or more γH2AX foci was calculated. Neurons with pan-nuclear γH2AX signals were excluded from the analysis.

For Cohort 2, double-immunolabeling studies were performed on free-floating 40-μm vibratome sections from the mid-frontal cortex (Brodmann area 46) and hippocampus as described [87]. Briefly, sections were washed with phosphate-buffered saline (PBS), permeabilized for 20 min in PBS containing 1% Triton X-100 (Sigma-Aldrich), and blocked with 10% horse serum in PBS for 1 h at room temperature. Sections were then incubated at 4 °C overnight with primary antibodies against γH2AX or 53BP1 in combination with antibodies against NeuN or GFAP (Additional file 1: Table S3). Fluorescein isothiocyanate (FITC)-conjugated secondary antibodies (1:75, Vector Laboratories) were used to detect anti-γH2AX, in which case anti-NeuN and anti-GFAP were detected with the Tyramide Signal Amplification (TSA) Direct (Red) system (NEN Life Sciences, Boston, MA). Anti-53BP1 was detected with the tyramide red system, in which case FITC-coupled secondary antibodies were used to detect anti-NeuN and anti GFAP. For the TSA step, horseradish peroxidase (HRP)-coupled secondary antibody was used at 1:500 for 30 min, and TSA working solution was used at 1:100 for 10 min. Negative controls included the omission of each primary antibody. Sections from the mid-frontal cortex and hippocampus (one each per case) were processed simultaneously under the same conditions, and experiments were performed twice. For some sections, Hoechst 33342 was used to stain nuclei. Sections were mounted onto Superfrost Plus slides (Fisher) and covered with ProLong Gold Antifade Mountant (Thermo Fisher Scientific).

Confocal images were acquired with a Radiance 2000 laser scanning confocal microscope (Bio-Rad, Hercules, CA) equipped with an Eclipse E600FN microscope (Nikon, Japan) and a Nikon Plan Apo 60x oil objective (NA 1.4; oil immersion). Two sections were analyzed per case. From each double-labeled section, a total of 10 images (1024 × 1024 pixels, final magnification 630x) were obtained from the mid-frontal cortex (layers 2–3) and hippocampus (CA1, CA3 and dentate gyrus) and analyzed using the ImageJ program [93] as described [87] to determine the proportion of NeuN- or GFAP-positive cells with γH2AX foci or 53BP1 labeling.

### Immunoperoxidase staining to assess AD pathology, calbindin and 53BP1 in human brain sections

For Cohort 2, free-floating 40-μm vibratome sections from the mid-frontal cortex (Brodmann area 46) and anterior hippocampus were washed with TBS, pre-treated in 3% H_2_O_2_, and blocked with 10% serum (Vector Laboratories, Burlingame, CA), 3% bovine serum albumin (Sigma-Aldrich), and 0.2% gelatin in TBST. Sections were incubated at 4 °C overnight with the following primary antibodies: anti-Aβ, anti-phosphorylated tau, anti-calbindin, or anti-53BP1 (Additional file 1: Table S3). Sections were then incubated in secondary antibodies (1:75, Vector Laboratories), followed by HRP-labeled Avidin D (ABC Elite, Vector Laboratories), and reacted with diaminobenzidine (DAB, 0.2 mg/ml) in 50 mM Tris (pH 7.4) and 0.001% H_2_O_2_. Control experiments included replacement of primary antibodies with pre-immune rabbit serum or non-immune IgG1, and preadsorption of the anti-53BP1 antibody at 4 °C overnight with a 20:1 excess of a synthetic 53BP1 peptide (Abcam, ab98293) designed to block activity of this antibody, followed by centrifugation at 5000 x g for 10 min and application of the supernatant to sections.

Immunostained sections were imaged with a BX50 digital microscope (Olympus). Aβ deposits and intraneuronal accumulations of phosphorylated tau were quantified as described [87]. Levels of calbindin and 53BP1 immunoreactivity were quantified using the Image-Pro Plus program (Media Cybernetics, Silver Spring, MD). For each case, 2 sections and 10 digital images per section (at 400x) were analyzed to estimate the average intensity of calbindin immunostaining (corrected optical density) in the dentate gyrus and the average number of 53BP1-immunoreactive cells per 0.1 mm^2^ in the mid-frontal cortex.

### Mice and treatments

Male and female C57Bl/6 J mice were maintained on a 12 h light/dark cycle. Experiments were conducted during the light cycle. Mice had free access to food (PicoLab Rodent Diet 20, LabDiet) and water and were housed 2–5 per cage. All studies were approved by the UCSF Institutional Animal Care and Use Committee and were consistent with NIH guidelines. All mice used were wildtype.

Some mice were exposed once to whole body ionizing radiation (4 or 8 Gy) from a Mark-I Cesium^137^ source (JL Shepherd and Associates, San Fernando, CA) in a partitioned clear plastic chamber on a rotating platform. The exposure took 1.16–3.32 min and mice were killed between 15 and 120 min after irradiation as indicated in figure legends.

Unless indicated otherwise, mice were killed following anesthesia with Avertin (tribromoethanol, 250 mg/kg) by transcardial perfusion with 0.9% NaCl. Brains were dissected, divided along the sagittal midline, and hemibrains were frozen on dry ice in cryovials (Wheaton) or fixed in 4% paraformaldehyde in PBS.

For treatment with kainate alone, kainate was dissolved in 0.9% saline to a concentration of 4 mg/ml. Some mice received a single intraperitoneal injection of kainate (20 or 30 mg/kg body weight) or saline. One hour after the injection, mice were anesthetized with Avertin (tribromoethanol, 250 mg/kg) and perfused transcardially with 0.9% NaCl.

For use in combination with irradiation experiments, kainate was dissolved in 0.9% saline to a concentration of 2 mg/ml. All mice received a single intraperitoneal (i.p.) injection of kainate (20 mg/kg body weight) or saline. Some of the mice were then placed into a partitioned clear plastic chamber and received 8 Gy whole body ionizing radiation 15 min after the i.p. injection. They were killed 60 min after the irradiation. Non-irradiated mice were killed 75 min after the i.p. injection. All mice injected with kainate displayed signs of epileptic activity ranging from rhythmic mouth and facial movements to full motor seizures with loss of postural control.

### Immunohistochemical analysis of mouse brain tissues

Hemibrains were drop-fixed with 4% paraformaldehyde in PBS for 48 h at 4 °C, washed in PBS, and infiltrated with 30% sucrose in PBS until hemibrains sank (≥ 12 h). Hemibrains were then cut into 30 μm sections with a SM200R sliding microtome (Leica). Sections were stored at − 20 °C in a solution of 40% PBS, 30% glycerol and 30% ethylene glycol until further use.

For fluorescence immunohistochemistry, sections were washed in TBS, permeabilized in TBS with 0.5% Triton X-100 (Sigma-Aldrich), and incubated for 15 min at 110 °C in citrate buffer (pH 6.0) for antigen retrieval. After incubating sections for 15 min in 3% H_2_O_2_ and 10% methanol, sections were blocked in 10% goat serum in TBS for 1 h and then incubated overnight at 4 °C with antibodies against parvalbumin, γH2AX, NeuN, or 53BP1 (Additional file 1: Table S3) in 3% goat serum in TBS with gentle rocking. The next day, sections were rocked at room temperature for 2 h, washed in TBS with 0.1% Tween and incubated for 2 h at room temperature with Alexa Fluor secondary antibodies (Thermo Fisher Scientific) at 1:500–1:2000 dilution in TBS with 3% goat serum. Sections were mounted onto glass slides and covered with ProLong Diamond Antifade Mountant (Thermo Fisher Scientific).

Confocal images were acquired from hippocampal CA1 or dentate gyrus with an LSM 880 confocal laser scanning microscope (Zeiss) and a 20x objective lens (NA 0.8). Z-stack images were taken with sequential acquisition settings at pixel size of 1.66 μm for low-magnification images and 0.21 or 0.41 μm for high-magnification images. Maximum intensity projections were created for all images. Images of the entire dentate gyrus were stitched together using ZEN software (Zeiss). Images acquired from two coronal sections per mouse were analyzed. Briefly, the dentate gyrus and a background area containing no cells were manually traced as regions of interest. After subtracting the γH2AX fluorescence intensity of the background region from the fluorescence intensity in the dentate gyrus, the fluorescence intensity in kainate-injected mice was normalized to that in saline-injected mice.

### DI-PLA on mouse brain sections

Snap-frozen mouse hemibrains were embedded in O.C.T. compound (Tissue-Tek), cryosectioned at 10 μm, mounted on Superfrost Plus Gold slides (Thermo Fisher Scientific), and stored at − 80 °C until use. Using the Duolink PLA Orange detection reagent (Sigma-Aldrich) per manufacturer’s instructions, the DI-PLA was then carried out as described [23] with the following modifications. Before blunting DSB ends, endogenous biotin was blocked with a biotin blocking kit (Molecular Probes) per manufacturer’s instructions. Ligation of the biotinylated linker was carried out for 1 h at room temperature as opposed to overnight at 16 °C. Sections were incubated in anti-γH2AX and anti-biotin antibodies in PBS with 0.1% Tween and 5% goat serum overnight at 4 °C. After completion of PLA chemistry per manufacturer’s instructions, sections were incubated with anti-NeuN antibody in PBS with 0.1% Tween and 5% goat serum for 0.5–1.0 h at room temperature. Sections were then incubated with Alexa Fluor-conjugated secondary antibodies (Thermo Fisher Scientific; 1:1000) in PBS with 0.1% Tween and 5% goat serum for 1 h at room temperature to visualize NeuN in addition to the PLA signal. Sections were then washed in PBS with 0.1% Tween and then with PLA wash buffers A and B, prepared per manufacturer’s protocols. After completion of the DI-PLA protocol, sections were allowed to air dry at room temperature while protected from light. They were then covered with ProLong Diamond Antifade Mountant (Thermo Fisher Scientific).

Slides were imaged with a Zeiss LSM 880 confocal microscope and a 20X objective (NA 0.8). Z-stack images were taken with sequential acquisition settings at pixel sizes of 0.15–0.41 μm. Maximum intensity projections were created for all images. As specified in figure legends, 3–4 images of the somatosensory cortex or dentate gyrus were obtained from each of two sections per mouse. For negative control sections, 2–3 images were obtained from a single section per mouse.

Images were analyzed using ImageJ [93] and a macro developed in-house. Briefly, maximum intensity projections were background subtracted, the NeuN channel was then thresholded and smoothed, and neuronal nuclei were identified and assigned as regions of interest (ROI) using the “analyze particles” function. The “find maxima” function was then used on the corresponding DI-PLA channel to automatically count the number of DI-PLA foci in the defined ROI. For cortical sections, the number of DI-PLA foci per neuron was calculated; for dentate gyrus sections, the number of DI-PLA foci per 100 μm^2^ neuronal area was calculated. A background noise tolerance was set by the experimenter to ensure only DI-PLA foci were detected as “maxima.” For any given experiment, all user-defined variables were kept constant across all images. Calculations were processed using Python scripts developed in-house.

### Generation, treatment and immunocytochemistry of primary neuronal cultures

Primary hippocampal neuronal cultures were established from individual day-0 C57Bl/6 J mouse pups as described [75]. Cells were cultured on poly-D-lysine-coated 96-well plates (Greiner; 20 wells/pup) in 100 μl Neurobasal medium (Thermo Fisher Scientific) supplemented with 2% B27 (Thermo Fisher Scientific), penicillin/streptomycin, and 1x GlutaMAX Supplement (Thermo Fisher Scientific). On days in vitro (DIV) 7, 100 μl of fresh medium was added to the conditioned medium in each well.

At DIV 14, cultures were pretreated for 1 h with fully conditioned medium containing vehicle or 1 μM tetrodotoxin (TTX) and then treated for 1 h with the same conditioned medium containing vehicle or bicuculline (10 μM). Cultures were then fixed with 4% paraformaldehyde in PBS for 15 min.

Fixed neuronal cultures were permeabilized in PBS with 0.2% Triton X-100 (Sigma-Aldrich) for 5 min, blocked in 10% goat serum in PBS for 1 h, and incubated with anti-γH2AX (1:1000) and anti-NeuN (1:4000) antibodies in 2% goat serum for 1.5 h at room temperature. After 5 brief washes in PBS, cultures were incubated with Alexa-Fluor conjugated secondary antibodies (1:2000, Thermo Fisher Scientific) and Hoechst 33342 (5 μM) in PBS with 2% goat serum for 1 h at room temperature. After 5 more brief washes in PBS, cultures were kept in PBS at 4 °C until imaging.

Immunostained cultures were imaged with a high-content ArrayScan system (Thermo Fisher Scientific) and a 40x objective, yielding 1024 × 1024 pixels per image. For each well, 15–20 images were obtained from different areas. For the γH2AX channel, maximum intensity projections were calculated from 4 images taken at 1 μm intervals along the z-axis (2 above and 2 below the focal plane). For all other channels, a single z-plane was imaged. ArrayScan software was used for image analysis. Briefly, the Hoechst 33342 channel was used to define nuclei as regions of interest and intensity values were calculated within those nuclei for each channel. An average nuclear γH2AX intensity was calculated for NeuN-positive cells. The median γH2AX intensity for all neurons in a well was calculated. For each independent neuronal culture, all wells of comparable conditions were treated as technical replicates. The means of technical replicates were then calculated and independent cultures were used as biological replicates for statistical analyses.

### Comet assay (single cell gel electrophoresis)

Primary hippocampal neuronal cultures were treated with bicuculline (5 μM), etoposide (10 μM) or vehicle for 1 h at DIV 14. Cultures were rinsed once with cold Ca^2+^ and Mg^2+^-free PBS, collected by gentle scraping, spun down, and mixed with low-melting point agarose (Trevigen). 50 μl of the mixture was applied onto each CometSlide (Trevigen) and allowed to solidify at 4 °C for 30 min. Lysis and comet assay were performed according to instructions provided with the CometAssay kit (Trevigen) with minor modifications. Briefly, cells were lysed for 30 min at 4 °C, immersed in 1x TBE buffer for 15 min, and electrophoresis was performed at neutral pH at 1 V/cm (measured electrode to electrode) for 30 min. Slides were immersed in dH_2_O for 5 min, 70% ethanol for 5 min, and dried at room temperature overnight. Samples were then stained with SYBR Green and imaged with a Zeiss LSM 880 microscope and a Fluar 5X objective (NA 0.25). DNA damage was assessed by measuring comet tail length using OpenComet software (v1.3.1). Experiments were performed in three independent cultures established from different mice. A total of 150–200 nuclei were measured for each mouse and treatment condition.

### Statistical analysis

Biological units were randomized during assays, imaging and analyses. All images for a given experiment were acquired using the same acquisition parameters. Wherever possible, investigators were blinded during image acquisition and quantitations to clinicopathologic group of humans and to treatment of mice. After acquisition, images for a given experiment were randomized and renamed using a custom MATLAB (MathWorks) script. Data were formatted and processed in Excel (Microsoft), MATLAB (MathWorks) or Python. Statistical analyses were carried out in Prism (GraphPad). For hypothesis testing, data were first tested for normality using the Shapiro-Wilk test. For normally distributed data, unpaired two-tailed Student’s t-tests were used to compare two groups, and one-way or two-way ANOVA with Holm-Sidak post-hoc tests to compare three or more groups. For data that were not normally distributed, we used the Mann-Whitney U test to compare two groups and the Kruskal-Wallis test and Holm-Sidak post-hoc tests to compare three or more groups. The null hypothesis postulated no difference between groups and was rejected at *P* < 0.05. Quantitative data are presented as means ± SEM.

## Results

### AD and MCI are associated with an accumulation of γH2AX foci in nuclei of neurons and astrocytes

To explore whether AD is associated with evidence for excessive neuronal DSBs, we first obtained *postmortem* brain tissues from a relatively small pilot cohort (Additional file 1: Table S1) and co-labeled sections of the orbitofrontal cortex with antibodies against γH2AX and the postmitotic neuronal marker NeuN. In this cohort, we detected a trend towards increased proportions of neurons with γH2AX foci and an increased number of γH2AX foci per neuron in AD and mild cognitive impairment (MCI) cases as compared to cases without cognitive impairment (Additional file 1: Figures S1 and S2a). Consistent with a previous report [76], several AD cases and one MCI case had high levels of γH2AX immunoreactivity in a proportion of NeuN-negative cells (Additional file 1: Figure S3 and data not shown), whereas the control cases did not. However, this non-neuronal γH2AX immunostaining was pan-nuclear rather than focal and, thus, may not represent DSBs (see below).

**Fig. 1:**
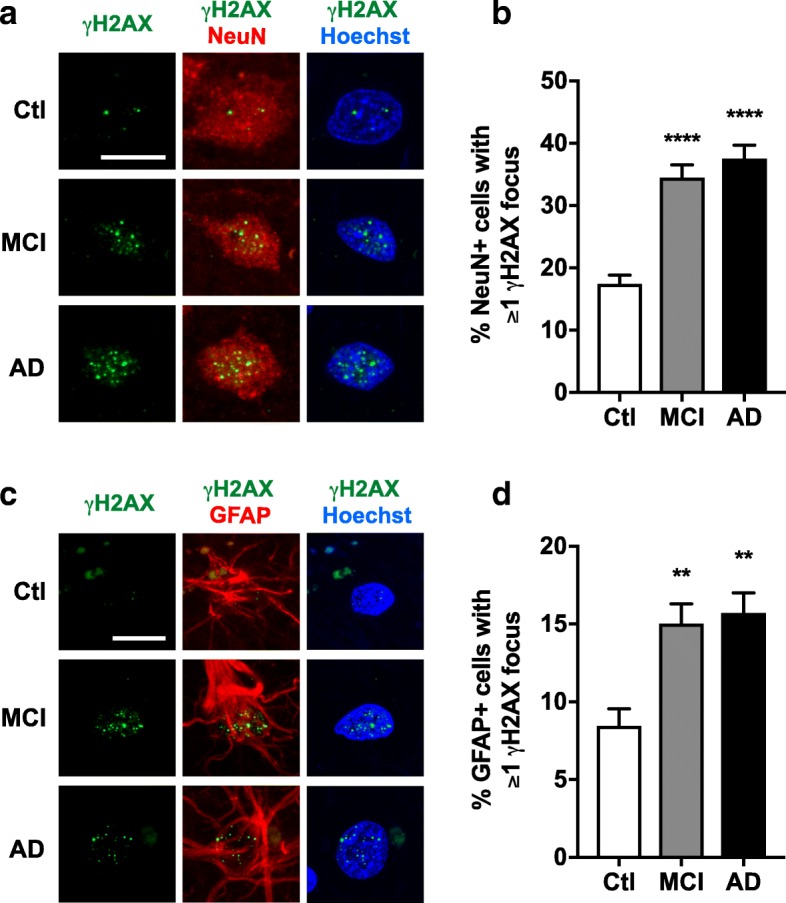
Increased proportion of neurons and astrocytes with γH2AX foci in frontal cortex of cases with MCI or AD. Frontal cortex sections from cognitively unimpaired controls (Ctl) and from MCI and AD cases were co-labeled for γH2AX (green) and for NeuN (red) (**a**, **b**) or the astroglial marker GFAP (red) (**c**, **d**). Nuclei were stained with Hoechst 33342 (blue). **a** Representative confocal images of neuronal γH2AX staining. **b** Proportion (%) of neurons with at least one γH2AX focus. **c** Representative confocal images of astroglial γH2AX staining. **d** Proportion (%) of astrocytes with at least one γH2AX focus. *n* = 8 Ctl, 7 MCI, and 8 AD cases from Cohort 2. **p < 0.01, ****p < 0.0001 vs. Ctl by one-way ANOVA and Holm-Sidak test. Scale bars: 10 μm. Bar graphs represent means ± SEM

Based on these preliminary findings, we extended our analysis to an independent, larger cohort of human cases (Additional file 1: Table S2). We found that both AD and MCI cases had twice as many neurons with γH2AX foci in the frontal cortex than cognitively unimpaired controls (Fig. 1a, b). The number of γH2AX foci per neuron was also higher in AD and MCI cases than in controls (Additional file 1: Figure S2b). Similar changes were observed in the hippocampal CA1 region (Additional file 1: Figure S4a).

AD and MCI cases showed comparable levels of neurons with DSBs (Fig. 1a, b, Additional file 1: Figure S4a), even though the accumulation of Aβ and phosphorylated tau was much more extensive in AD than MCI brains (Additional file 1: Figure S5). Interestingly, AD and MCI cases showed comparable levels of calbindin depletion in the dentate gyrus (Additional file 1: Figure S5), an abnormality that likely reflects aberrant neural network activity [81, 82], which has been implicated in the generation of neuronal DSBs [64, 106].

In Cohort 2, we also examined GFAP-positive astrocytes in greater detail. Compared to controls, AD and MCI cases had an increased proportion of GFAP-positive astrocytes with γH2AX foci in the frontal cortex (Fig. 1c, d) and CA1 region (Additional file 1: Figure S4b). Thus, our data suggest that AD causes an accumulation of DSBs in both neurons and astrocytes and that this process begins at relatively early stages of disease progression.

### AD and MCI are associated with neuronal increases and astroglial decreases in pan-nuclear 53BP1 staining

**Fig. 2:**
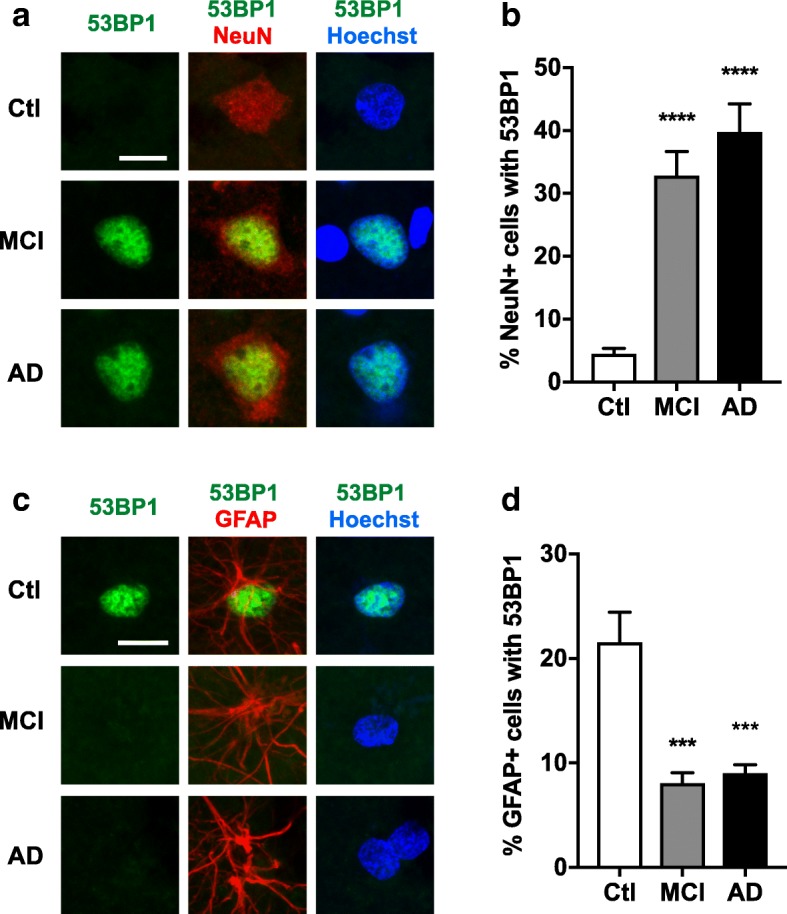
Pan-nuclear 53BP1 staining is increased in neurons and decreased in astrocytes in frontal cortex of MCI and AD cases. Frontal cortex sections from cognitively unimpaired controls (Ctl) and from MCI and AD cases were double-labeled for 53BP1 (green) and for NeuN (red) (**a, b**) or the astroglial marker GFAP (red) (**c**, **d**). Nuclei were stained with Hoechst 33342 (blue). **a** Representative confocal images of neuronal 53BP1 staining. **b** Proportion (%) of neurons with pan-nuclear 53BP1 staining. **c** Representative confocal images of astroglial 53BP1 staining. **d** Proportion (%) of astrocytes with pan-nuclear 53BP1 staining. *n* = 8 Ctl, 7 MCI, and 8 AD cases from Cohort 2. ***p < 0.001, ****p < 0.0001 vs. Ctl by one-way ANOVA and Holm-Sidak test. Scale bars: 10 μm. Bar graphs represent means ± SEM

Eliciting neuronal DSBs by irradiation or other means in mice leads to the formation of distinct γH2AX foci in brain cells that are co-labeled by antibodies to 53BP1 (Additional file 1: Figure S6 and ref. [106]), a protein that is rapidly recruited to DSBs and has many putative functions, including the promotion of DSB repair by the non-homologous end-joining pathway [84]. To further support our conclusion that the γH2AX foci we detected in AD and MCI cases represent genuine DSBs, we immunostained brain sections from Cohort 2 for this additional DSB marker. AD and MCI cases showed marked and comparable increases in the proportion of neurons with nuclear 53BP1 staining in the frontal cortex and CA1 region (Fig. 2a, b, Additional file 1: Figure S4c). However, in most neurons this 53BP1 staining was diffuse and pan-nuclear rather than focal or punctate (Fig. 2a), similar to what others have observed in neurons of deep cerebellar nuclei from AD cases [10]. To ensure the target specificity of this staining, we tested an independent 53BP1 antibody. It yielded a similar staining pattern, which was eliminated by pre-adsorption of the antibody with a peptide representing the 53BP1 immunogen (Additional file 1: Figure S7).

To investigate whether the increased neuronal 53BP1 staining in AD and MCI cases might have emerged *postmortem*, we elicited neuronal DSBs in mice by irradiation and drop-fixed their brains in 10% neutral buffered formalin for 48 h either immediately after sacrifice or after different *postmortem* intervals (PMI). Even after a PMI of 9 h, irradiated mice showed marked increases in crisp 53BP1 foci, which co-localized with γH2AX, rather than pan-nuclear increases in diffuse 53BP1 staining (Additional file 1: Figure S8). These results make it unlikely that the increases in neuronal 53BP1 labeling we and others [10] have detected in humans with AD or MCI represent a *postmortem* artifact.

Surprisingly, AD and MCI cases had a lower proportion of GFAP-positive astrocytes with pan-nuclear 53BP1 staining than controls (Fig. 2c, d, Additional file 1: Figure S4d). Although the processes that cause AD-associated changes in 53BP1 expression remain to be determined, our results make it likely that they affect neurons and astrocytes differentially.

### Pan-nuclear H2AX phosphorylation in neurons can be caused by increases in neuronal activity but is unlikely to represent DSBs

**Fig. 3:**
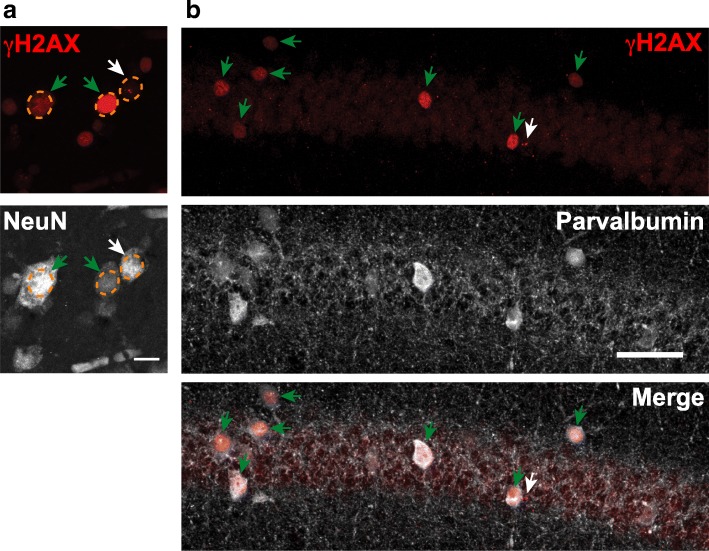
Pan-nuclear H2AX phosphorylation in neurons of humans and mice. **a** Representative confocal microscopic images of sections from the orbitofrontal cortex of an AD case from Cohort 1. Sections were immunostained for γH2AX (red) and NeuN (grey). Orange circles outline nuclei. Two distinct patterns of γH2AX immunoreactivity were observed: foci (white arrows) and pan-nuclear (green arrows). Scale bar: 10 μm. **b** Representative confocal images of sections from the CA1 region of a mouse. Sections were immunostained for γH2AX (red) and parvalbumin (grey). Green arrows highlight the large proportion of parvalbumin-positive interneurons showing pan-nuclear γH2AX labeling under physiological conditions. Scale bar: 50 μm

As described above, focal γH2AX immunoreactivity is a well-established marked of DSBs. While measuring this marker, we noticed that γH2AX immunostaining labels the nuclei of some neurons diffusely, in humans as well as mice (Fig. 3). In wildtype mice that were not exposed to any experimental manipulation before death, a large proportion of specific neuronal populations showed prominent pan-nuclear γH2AX staining, for example, ≥30% of parvalbumin-positive interneurons in CA1 (Fig. 3b). These findings raise the intriguing possibility that focal and pan-nuclear patterns of H2AX phosphorylation have different causes and consequences.

**Fig. 4:**
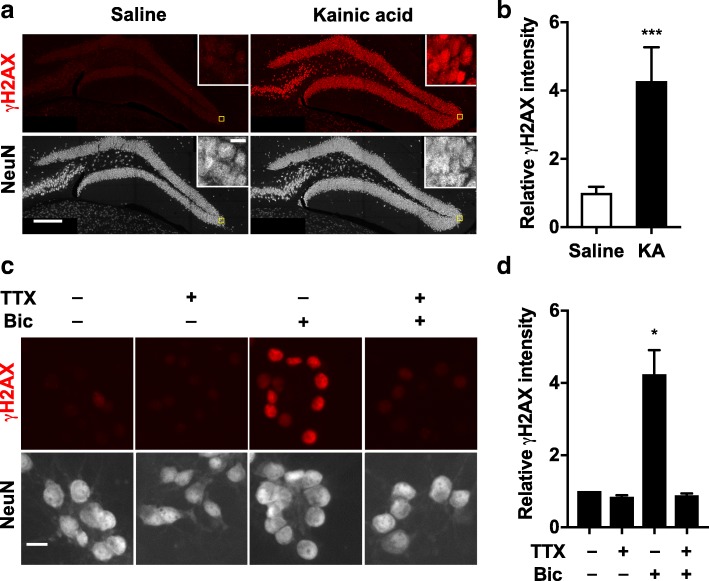
Increased neuronal activity causes pan-nuclear neuronal γH2AX labeling in vivo and in vitro. **a**, **b** Mice were analyzed 1 h after they received an intraperitoneal injection of kainate (KA, 20 or 30 mg/kg) or vehicle (saline) at 4–6 months of age. **a** Representative confocal microscopic images of dentate gyrus sections co-labeled for γH2AX (red) and NeuN (grey). Insets show higher magnification views of the areas outlined by yellow squares. Note the pan-nuclear pattern of the γH2AX labeling. Scale bars: 200 μm; 10 μm (insets). **b** Quantitation of γH2AX immunofluorescence intensity in the dentate gyrus. Mean levels in saline-treated mice were arbitrarily defined as 1.0. *n* = 9 mice per group (pooled from 3 independent experiments). ***p < 0.001 by Mann-Whitney test. **c**, **d** Primary hippocampal neuronal cultures from mice were pretreated for 1 h with vehicle or tetrodotoxin (TTX, 1 μM) on DIV 14. Vehicle or bicuculline (Bic, 10 μM) was then added to the medium and cultures were incubated for 1 h. **c** Representative widefield images of neuronal cultures co-labeled for γH2AX (red) and NeuN (grey). Scale bar: 10 μm. **d** Quantitation of neuronal γH2AX immunofluorescence intensity. For each culture, the mean levels in different wells of saline-treated cultures were arbitrarily defined as 1.0. *n* = 4 independent cultures from different mice per condition. *p < 0.05 vs. mean of 1.0 (control) by one sample t-test. Bars represent means ± SEM

Because parvalbumin-positive interneurons are among the fastest spiking neurons in the brain [32], we wondered whether neuronal activity itself could increase pan-nuclear γH2AX staining. To test this hypothesis, we injected mice with the glutamate receptor agonist kainate to induce epileptic activity. Within one hour after mice received an intraperitoneal injection of kainate (20–30 mg/kg body weight), they all showed evidence of seizure activity (data not shown). Compared to saline-injected controls, kainate-injected mice showed a marked increase in pan-nuclear neuronal γH2AX staining in the dentate gyrus (Fig. 4a, b).

To further assess the relationship between neuronal activity and pan-nuclear γH2AX staining, we increased the activity of mouse neurons by treating primary cultures with the GABA_A_ receptor antagonist, bicuculline. This treatment was well tolerated and did not cause overt toxicity at the doses used here (data not shown). One hour after bicuculline treatment, NeuN-positive neurons showed a robust increase in γH2AX staining, which was pan-nuclear rather than focal (Fig. 4c, d). Pre-treatment of neuronal cultures with the sodium channel antagonist tetrodotoxin (TTX) abolished the bicuculline-induced increases in γH2AX staining, confirming that they were indeed activity-dependent (Fig. 4c, d). Taken together, these results suggest that high levels of neuronal activity promote pan-nuclear H2AX phosphorylation in vitro and in vivo, possibly without causing the formation of DSBs.

### Comet assay and DI-PLA reveal no evidence for DSBs in neurons with pan-nuclear H2AX phosphorylation

**Fig. 5:**
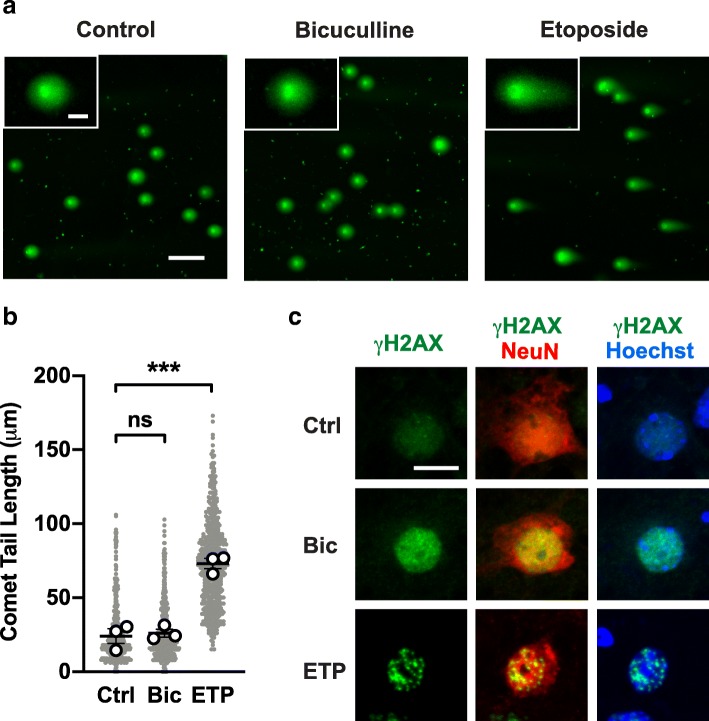
Neutral comet assay detects no DSBs in primary cultures with elevated neuronal activity and pan-nuclear γH2AX elevation. **a**–**c** Cultures of primary hippocampal mouse neurons were assessed for DSB levels by the neutral comet assay (**a**, **b**) or immunocytochemistry (**c**). Cells were treated with vehicle (Ctrl), 10 μM bicuculline (Bic) or 5 μM etoposide (ETP) for 1 h before harvesting. **a** Representative images of cell nuclei stained with SYBR Green after electrophoresis at neutral pH. Insets show higher magnification views. Scale bar: 100 μm; 20 μm (insets). **b** Quantitation of comet tail lengths. A total of 150–200 nuclei were measured for each mouse and condition. *n* = 3 cultures/mice per condition. Circles indicate mean values for each mouse; grey dots indicate individual nuclei. ***p < 0.001 by one-way ANOVA and Holm-Sidak test. ns, not significant. **c** Representative confocal images of cultured neurons exposed to the indicated treatments. Cells were co-labeled for γH2AX (green), NeuN (red), and Hoechst 33342 (blue). Scale bar: 10 μm

To determine whether pan-nuclear H2AX phosphorylation is associated with DSBs, we first performed the comet assay in primary hippocampal cultures. Performed at neutral pH, this assay mainly detects DSBs, the extent of which is reflected by the length of comet tails that emerge from cell nuclei upon electrophoresis [78]. The mean comet tail length from bicuculline-treated samples was not significantly different from mock-treated samples (Fig. 5a, b), although bicuculline treatment induced pan-nuclear γH2AX staining (Fig. 5c). Positive control samples treated with the genotoxic agent etoposide showed increased comet tail length and γH2AX foci (Fig. 5). These results further support the notion that pan-nuclear γH2AX formation can occur in the absence of DSBs.

**Fig. 6:**
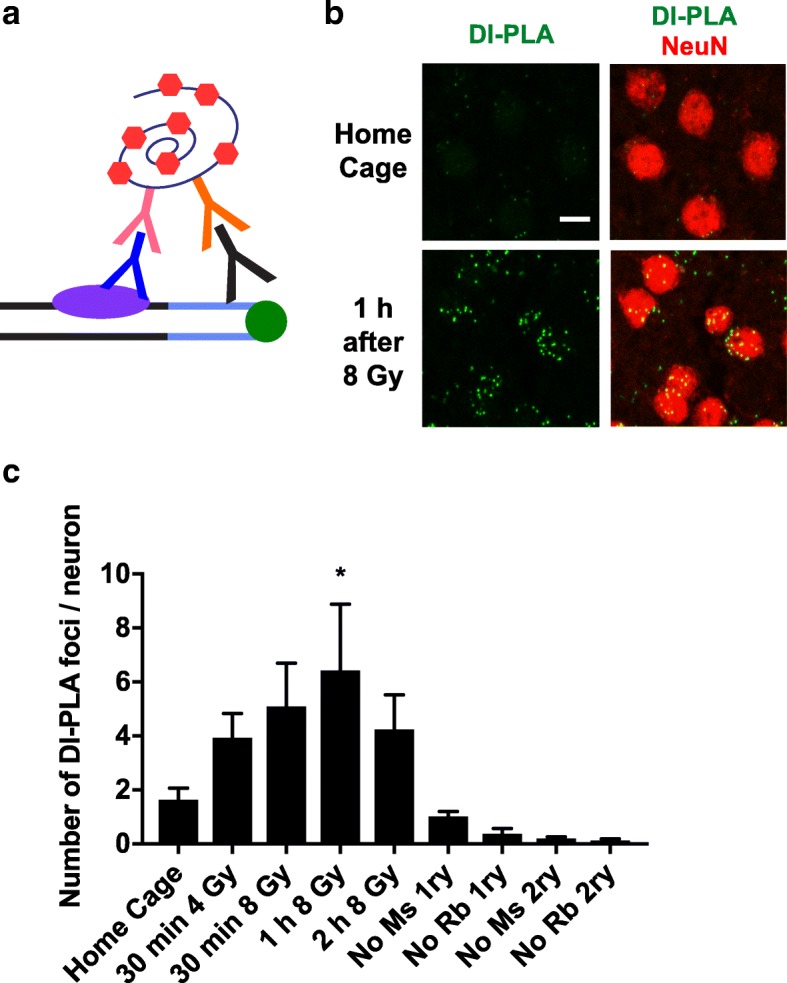
DI-PLA detects DSBs in neurons of mice exposed to ionizing radiation. **a** Schematic of the DI-PLA. A biotinylated (green circle) linker (light blue) is ligated to the free ends of a DSB (black lines). An antibody (black) against biotin and an antibody from a different species (dark blue) against a DSB-associated repair factor (purple oval) are then added. Species-specific secondary antibodies (pink and orange) conjugated to DNA oligonucleotides are used to detect the primary antibodies. If the primary antibody targets are located in close proximity, a connector oligonucleotide ligates them to one another and allows for the creation of a closed circular DNA template that can be amplified by rolling circle amplification. Fluorescently labeled oligonucleotides (red hexagons) hybridize to the amplicon and can be visualized by fluorescence microscopy. **b** Representative confocal images of DI-PLA signals and NeuN immunostaining in somatosensory cortex sections from 3 to 6-month-old mice that were untreated (Home Cage, top) or exposed to 8 Gy whole-body ionizing radiation 1 h before analysis (bottom). DI-PLA single channel (left) and merged views (right) are shown. Scale bar: 10 μm. **c** Number of DI-PLA foci per neuron in somatosensory cortex from mice analyzed at different time points after 4 Gy or 8 Gy irradiation. Besides unirradiated mice, negative controls included the omission of mouse (Ms) or rabbit (Rb) primary antibodies (1ry) and of mouse or rabbit secondary antibodies (2ry). *n* = 4–5 mice per group. *p < 0.05 vs. Home Cage by one-way ANOVA and Holm-Sidak test. Bars represent means ± SEM

The neutral comet assay does not determine the specific cell types that harbor DSBs. To address this shortcoming and to further differentiate between DSBs and pan-nuclear γH2AX formation, we used the recently developed DNA damage in situ ligation followed by proximity ligation assay (DI-PLA) [23]. This assay (Fig. 6a) builds on the original findings that double stranded linkers can be specifically ligated to free DSBs in situ [29] and that when such linkers are biotinylated, DSB ends can be purified on streptavidin beads [14]. To identify bona fide DSBs, Galbiati and colleagues combined this biotinylated DSB linker strategy with a proximity ligation assay that allows for the visualization of chromatin sites at which free DSB ends are located in close proximity to γH2AX [23].

We first confirmed that the DI-PLA readily detects DSBs in the brains of mice that were exposed to whole-body ionizing radiation (Fig. 6b). Following this treatment, DI-PLA signals in neurons increased, peaking at approximately 1 h after irradiation, and then decreased, most likely as the result of DSB repair (Fig. 6c). Negative controls included the omission of the biotinylated linker and of primary or secondary antibodies (Fig. 6c).

**Fig. 7:**
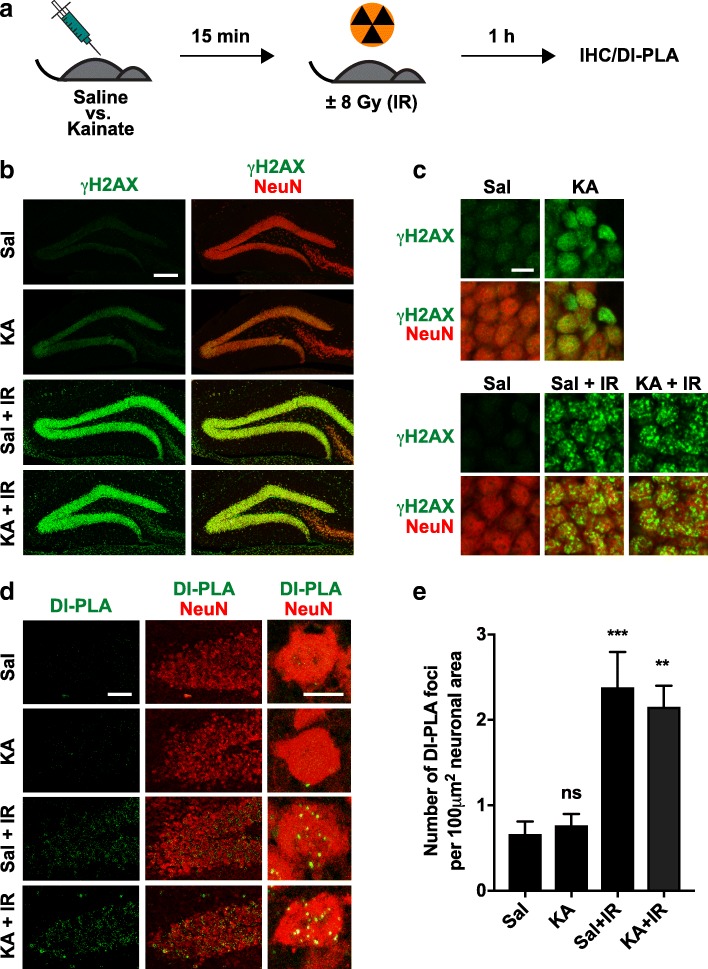
Experimental differentiation between focal and pan-nuclear neuronal γH2AX labeling. **a** Schematic of experimental design. Mice received an intraperitoneal injection of kainate (KA, 20 mg/kg) or saline (Sal) at 3–6 months of age. Fifteen minutes later, roughly half of them were exposed to 8 Gy ionizing radiation (IR). One hour later, all mice were sacrificed and their brains processed for fluorescence immunohistochemistry (**b**, **c**) and DI-PLA (**d**, **e**). **b** Representative confocal images of dentate gyrus sections co-labeled for γH2AX and NeuN. γH2AX single channel (left) and merged views (right) are shown. Scale bar: 200 μm. **c** Representative higher magnification views of sections similar to those in (b). Scale bar: 10 μm. **d** Representative confocal images from the apex of the dentate gyrus showing DI-PLA signals (left) and merged views with NeuN co-labeling (middle). High-magnification images (right) show individual neurons with DI-PLA signals (green) and NeuN immunostaining (red). Scale bar: 50 μm (left and middle), 10 μm (right). **e** Number of DI-PLA foci per 100 μm^2^ neuronal area in the apex of the dentate gyrus. *n* = 4–7 mice per group. Two-way ANOVA revealed an effect of irradiation (*p* < 0.0001) but not of kainate (*p* = 0.8093) and no interaction between them (*p* = 0.5201). **p < 0.01, ***p < 0.001 vs. Sal by Holm-Sidak test. Bars represent means ± SEM

To exclude the possibility that pan-nuclear γH2AX staining simply obscures the presence of γH2AX foci representing DSBs, we designed the experiment outlined in Fig. 7a. To increase the number of neurons with pan-nuclear γH2AX staining, mice received an intraperitoneal injection of kainate (20 mg/kg), or saline as a negative control. To cause DSBs, roughly half of the mice received 8 Gy of ionizing whole-body irradiation shortly after the injection of kainate or saline. All mice were then analyzed by immunohistochemistry and DI-PLA. Kainate treatment increased the proportion of neurons with pan-nuclear γH2AX staining in the dentate gyrus but did not cause γH2AX foci, unless mice were also irradiated (Fig. 7b, c). The dose of irradiation used here caused many neuronal DSBs, which were readily detectable by γH2AX staining and by DI-PLA independent of whether neurons had kainate-induced increases in pan-nuclear γH2AX staining or not (Fig. 7d, e). Thus, in the absence of focal γH2AX staining, pan-nuclear γH2AX staining should not be interpreted as evidence for DSBs and may reflect increased levels of neuronal activity.

Lastly, we attempted to use the DI-PLA for the detection of DSBs in human *postmortem* tissues. However, we observed substantial signal variations when we analyzed sections from the same cases on different occasions (data not shown). We did not find such variations in mice, in which the DI-PLA yielded reliable results even when we simulated human *postmortem* conditions (Additional file 1: Figure S9), suggesting that the difference may be due to *premortem* factors or other variables.

## Discussion

Our results suggest that AD causes neuronal accumulation of DSBs even at relatively early stages of the illness. Because unrepaired DSBs can lead to cell death [4, 120], neuronal DSB accumulation may contribute to neurodegeneration. DSBs can also cause changes in transcription from gene promoters located near break sites [36, 47, 85, 96], which could lead to changes in neuronal and synaptic functions. Recent evidence suggests that neuronal DSB formation could also provide entry points for the retro-insertion of genomic complementary DNAs and give rise to mosaic mutations of the *APP* gene in AD [49]. DSB-related changes in gene expression may explain, at least in part, why some brain regions that are relatively resistant to neuronal loss in AD—for example, the dentate gyrus—still show molecular and radiological alterations suggestive of major dysfunction [1, 2, 13, 81, 103].

In dividing cell lines, some experimental conditions can cause γH2AX foci without causing DSBs [43, 73, 88]. However, it is unclear how these findings relate to DNA damage responses in the brain. In vivo, γH2AX foci appear to be reliable indicators of true DSBs [54]. By immunostaining for γH2AX foci in *postmortem* brain sections, we showed that neuronal DSBs accumulate in MCI and AD to a greater extent than in cognitively normal controls.

How does AD increase DSBs in neurons? During normal aging, oxidative DNA damage accumulates in the promoters of genes involved in learning and memory and in neuronal survival [62]. AD further increases oxidative damage in neurons [8, 59, 60, 69]. Oxidative damage can result in DSBs, particularly when multiple oxidative lesions occur close to one another [94, 97]. Therefore, excessive production and/or reduced inactivation of free radicals represent plausible mechanisms for the accumulation of neuronal DSBs in AD. Other lines of evidence suggest that aberrant neuronal activity can increase neuronal DSBs independent of oxidative stress [106] and that increased or excessive stimulation of neuronal glutamate receptors can lead to DSB formation within the promoters of early-response genes [64]. In light of these studies, chronic network hyperexcitability, which is prevalent in AD [30, 48, 82, 111, 112], might also contribute to the accumulation of neuronal DSBs. In line with this notion, we found reduced neuronal levels of calbindin in the dentate gyrus of MCI and AD cases, a reliable indicator of chronic network hyperexcitability and excessive calcium influx [34, 72, 81]. However, acute kainate-induced seizure activity in mice resulted in pan-nuclear H2AX phosphorylation rather than DSB formation. This finding casts doubt on the role of acute epileptic activity as a potential cause of DSBs, although it cannot exclude the possibility that chronic epileptic activity or other types of network dysfunction contribute to DSB formation in AD and related conditions. Several recent studies have demonstrated an increase in the level of transposable elements in Alzheimer’s disease and other neurodegenerative tauopathies [26, 107]. As some transposons can cause DSBs through endogenous endonuclease activity [24], their increase might also contribute to the increase in DSBs we observed in MCI and AD.

Interestingly, AD patients show evidence for DNA repair deficits even in peripheral blood cells [11, 19] and several proteins with known roles in DSB repair are depleted in their brains [7, 41, 95, 98, 105]. Thus, deficits in DNA repair may also contribute to the abnormal neuronal accumulation of DSBs in AD. Several environmental factors, including viruses and a high fat diet, have been linked to the weakened DNA repair in AD. For example, several studies have found increased evidence for the presence of viral pathogens in AD brains, particularly herpes simplex viruses (HSV) and human herpesvirus 6 (HHV6) [9, 37, 52, 61, 89]. Infection with HSV1 can cause DNA damage in neurons, including DSBs [17], and impair DNA repair by NHEJ [17, 50, 86]. A recent study found that a high fat diet increased neuronal DSB levels and altered the balance of DSB repair pathways in aged APP/PSEN1 mice [118]. It is tempting to speculate that this process may contribute to the accelerated cognitive decline observed in APP/PSEN1 mice on this diet [104, 109, 114].

Because 53BP1 promotes the repair of DNA damage, including DSBs [38, 77, 116, 117], the absence of 53BP1-positive foci in neurons of MCI and AD cases with clear evidence for DSBs by γH2AX immunostaining in our study could, in principle, represent a DNA repair deficit in AD. Specifically, recruitment of 53BP1 to DSBs may be defective in AD. Such a recruitment defect would be consistent with a recent report of decreased 53BP1 foci at DSBs in models of *C9orf72* repeat expansion, a common cause of amyotrophic lateral sclerosis and frontotemporal lobar degeneration [113]. This decrease in 53BP1 foci was attributed, in part, to deficits in ubiquitin signaling [113], which is required for the maintenance of 53BP1 at DSBs [15, 40, 63, 66]. Abnormalities in ubiquitin-proteasome functions have also been detected in brains of patients with AD [44, 45, 56], which may explain why we did not find 53BP1 accumulation at DSBs in this condition. However, γH2AX foci in neurons of cognitively unimpaired controls also did not colocalize with 53BP1 (data not shown). This finding raises the possibility that 53BP1 fulfills different functions in humans than in mice, in which induction of DSBs consistently resulted in the formation of nuclear foci containing both γH2AX and 53BP1. It is unlikely that the absence of 53BP1 foci in human AD cases represents a *postmortem* artifact, as neuronal 53BP1 foci could be readily identified in brains of irradiated mice even after prolonged PMIs and when using similar tissue fixation conditions as in the human cases. It is also unlikely that AD depletes neuronal 53BP1 levels, as we found diffuse pan-nuclear increases in 53BP1 immunoreactivity in neurons of MCI and AD cases, as compared to controls.

In the process of quantifying DSBs in MCI and AD, we observed that γH2AX can also exist in a diffuse pan-nuclear pattern in neurons that is distinct from its focal accumulation at DSBs. We also found increases in pan-nuclear γH2AX in non-neuronal cells in AD brains, consistent with a previous report [76]. Similar pan-nuclear increases in γH2AX formation have been reported in the context of irradiation-induced apoptosis in resting human lymphocytes [18], in response to certain types of DNA damage in the absence of apoptosis in dividing cell lines and cultured primary cells [28, 74], after cellular infection of dividing cell lines with inactivated adeno-associated viral particles [21], and after treatment with a high-dose alkylating agent in a human amnion cell line [119].

Here, we were able to experimentally elicit pan-nuclear increases in γH2AX immunoreactivity in vitro and in vivo by increasing neuronal activity. In kainate-treated mice and in cultured neurons treated with bicuculline, most neurons with increased pan-nuclear γH2AX showed no evidence of DSBs by DI-PLA or neutral comet assay, suggesting that pan-nuclear and focal increases in γH2AX have different causes and functions. In line with this conclusion, low doses of the alkylating agent N-methyl-N′-nitro-N-nitrosoguanidine (MNNG) caused the formation of γH2AX foci and a positive neutral comet assay suggestive of DSBs in a dividing cell line, whereas higher doses of MNNG led to pan-nuclear H2AX phosphorylation without evidence for DSBs by neutral comet assay [119].

Because we detected particularly high levels of pan-nuclear γH2AX in fast-spiking interneurons at baseline, it is tempting to speculate that high levels of pan-nuclear γH2AX somehow support or result from high levels of physiological neuronal activity in vivo. Additional studies are needed to test these hypotheses and to unravel the mechanisms involved. Notwithstanding these uncertainties, our findings clearly demonstrate that increases in overall levels of neuronal γH2AX—as measured, for example, by western blot analysis—should not be interpreted as evidence for DSB accumulation, because such increases can result from changes in neuronal activity, and possibly other processes, in the absence of DSBs.

## Conclusions

Our study demonstrates that DSBs accumulate in neurons and astrocytes at early stages and during the progression of AD, a process that may contribute to neuronal dysfunction and degeneration. Further investigation of the causes and consequences of DSBs and other types of DNA damage in AD and related conditions may identify novel opportunities for therapeutic intervention.

## Additional file

Additional file 1: **Table S1.** Clinicopathological characteristics of human Cohort 1. **Table S2.** Clinicopathological characteristics of human Cohort 2. **Table S3.** Antibodies used in this study. **Figure S1.** Neuronal γH2AX labeling in frontal cortex of humans with or without MCI or AD. **Figure S2.** Neuronal γH2AX labeling in frontal cortex of humans with or without MCI or AD. **Figure S3.** Non-neuronal pan-nuclear H2AX phosphorylation in AD. **Figure S4.** Neuronal and astroglial γH2AX and 53BP1 labeling in the hippocampal CA1 region of cases with MCI or AD. **Figure S5.** Neuropathological alterations in human Cohort 2. **Figure S6.** Ionizing radiation causes focal accumulation of γH2AX and 53BP1 in neuronal nuclei of mice. **Figure S7.** Increased hippocampal 53BP1 staining in humans with MCI or AD. **Figure S8.** Increasing the *postmortem* interval (PMI) to 9 h does not change 53BP1 or γH2AX labeling of irradiation-induced DSBs in mouse brains. **Figure S9.** *Postmortem* delays in tissue processing and *postmortem* irradiation do not cause DI-PLA signals in mouse brains. (PDF 11500 kb)

## Abbreviations

AD: Alzheimer’s disease
Aβ: Amyloid-β
Bic: Bicuculline
Ctl: Control
DAB: Diaminobenzidine
DI-PLA: DNA damage in situ ligation followed by proximity ligation assay
DSBs: DNA double strand breaks
ETP: Etoposide
FITC: Fluorescein isothiocyanate
HHV6: Human herpesvirus 6
HRP: Horseradish peroxidase
HSV: Herpes simplex viruses
ICC: Immunocytochemistry
IHC: Immunohistochemistry
IR: Ionizing radiation
KA: Kainate
MCI: Mild cognitive impairment
MMSE: Mini-mental state examination
MNNG: N-methyl-N′-nitro-N-nitrosoguanidine
NHEJ: Nonhomologous end joining
PBS: Phosphate-buffered saline
PFA: Paraformaldehyde
PMI: *Postmortem* intervals
ROI: Regions of interest
Sal: Saline
TBS: Tris-buffered saline
TBST: Tris-buffered saline containing 0.1% Tween-20
TSA: Tyramide signal amplification
TTX: Tetrodotoxin
