# Supplementary material for: Early neuronal accumulation of DNA double strand breaks in Alzheimer’s disease
Source: Acta Neuropathol Commun. 2019 May 17;7:77. doi: 10.1186/s40478-019-0723-5 (PMC6524256; doi:10.1186/s40478-019-0723-5)
Supplement: Supplementary file 1 — Table S1. Clinicopathological characteristics of human Cohort 1. Table S2. Clinicopathological characteristics of human Cohort 2. Table S3. Antibodies used in this study. Figure S1. Neuronal γH2AX labeling in frontal cortex of humans with or without MCI or AD. Figure S2. Neuronal γH2AX labeling in frontal cortex of humans with or without MCI or AD. Figure S3. Non-neuronal pan-nuclear H2AX phosphorylation in AD. Figure S4. Neuronal and astroglial γH2AX and 53BP1 labeling in the hippocampal CA1 region of cases with MCI or AD. Figure S5. Neuropathological alterations in human Cohort 2. Figure S6. Ionizing radiation causes focal accumulation of γH2AX and 53BP1 in neuronal nuclei of mice. Figure S7. Increased hippocampal 53BP1 staining in humans with MCI or AD. Figure S8. Increasing the postmortem interval (PMI) to 9 h does not change 53BP1 or γH2AX labeling of irradiation-induced DSBs in mouse brains. Figure S9. Postmortem delays in tissue processing and postmortem irradiation do not cause DI-PLA signals in mouse brains. (PDF 11500 kb) [file 40478_2019_723_MOESM1_ESM.pdf]

Supplementary Information for

Early neuronal accumulation of DNA double strand breaks in Alzheimer's disease

Niraj M. Shanbhag[1,2†], Mark D. Evans[1†], Wenjie Mao[1†], Alissa L. Nana[2,3], William W. Seeley[2,3], Anthony Adame[4], Robert Rissman[4], Eliezer Masliah[4,5], and Lennart Mucke[1,2*]

[1]*Gladstone Institute of Neurological Disease, San Francisco, CA 94158, USA*
[2]*Memory and Aging Center, Department of Neurology, University of California San Francisco, San Francisco, CA 94158, USA*
[3]*Department of Pathology, University of California San Francisco, San Francisco, CA 94158, USA*
[4]*Department of Neurosciences, University of California at San Diego, La Jolla, CA 92093, USA*
[5]*Present address: Division of Neuroscience, National Institute on Aging, Bethesda, MD 20892, USA*

[†] These authors contributed equally to this study.

*Corresponding author
email – lennart.mucke@gladstone.ucsf.edu
tel – (415) 734-2504
fax – (415) 355-0131

This PDF file includes:
Tables S1 – S3
Figures S1 – S9

Table S1. Clinicopathological characteristics of human Cohort 1

Clinical Diagnosis	Age (years)	Gender	MMSE score	Braak stage	Duration (years)	PMI (hours)
Ctl	77	M	29	5	N/A	8.4
Ctl	76	M	29	2	N/A	8.2
MCI	77	M	28	2	ND	4.9
MCI	88	F	30	3	11	15.5
MCI	87	F	27	4	3	9.5
AD	69	F	27	6	9	12.9
AD	72	M	13	6	12	6.9
AD	63	M	7	6	6	17.8
AD	59	M	15	6	12	5.5
AD	65	F	18	6	11	10.9
AD	63	M	4	6	12	6
AD	80	M	23	6	9	6.5
AD	77	M	22	6	9	12.9
Ctl summary	77 ± 0.5	0% F	29 ± 0	3.5 ± 1.5	N/A	8.3 ± 0.1
MCI summary	84 ± 3.5	67% F	28.3 ± 0.9	3 ± 0.6	7 ± 4	10.0 ± 3.1
AD summary	69 ± 2.6	25% F	16.1 ± 2.8	6 ± 0	10 ± 0.8	9.9 ± 1.6

AD, Alzheimer’s disease; Ctl, control; MCI, mild cognitive impairment; MMSE, mini-mental state examination; N/A, not applicable; ND, not determined; PMI, *postmortem* interval. Summary data indicate means ± SEM, or percentage.

Table S2. Clinicopathological characteristics of human Cohort 2

Clinical Diagnosis	Age (years)	Gender	Blessed score	MMSE score	Braak stage	Duration (years)	PMI (hours)
Ctl	83	F	0	29	1	N/A	12
Ctl	51	M	3	26	0	N/A	12
Ctl	77	F	0	29	0	N/A	12
Ctl	73	F	0	30	1	N/A	12
Ctl	102	F	2	27	1	N/A	9
Ctl	93	F	1	30	1	N/A	18
Ctl	95	F	0	30	1	N/A	6
Ctl	83	F	2	30	1	N/A	12
MCI	86	M	1	29	5	1	4
MCI	90	F	1	24	3	1	12
MCI	93	F	1	21	1	2	6
MCI	84	M	1	28	1	5	8
MCI	91	F	1	29	3	11	4
MCI	80	F	0	29	1	1	12
MCI	86	M	6	25	2	1	4
AD	88	M	32	6	5	10	5
AD	68	M	33	13	6	10	19
AD	79	M	24	11	6	12	10
AD	76	M	24	13	5	10	6
AD	89	F	30	27	5	9	18
AD	88	F	19	16	6	12	12
AD	81	M	26	15	6	13	8
AD	87	M	26	8	6	8	7
Ctl summary	82 ± 5.6	88% F	1 ± 0.4	28.9 ± 0.5	0.8 ± 0.2	N/A	12 ± 1
MCI summary	87 ± 1.6	57% F	1.6 ± 0.8	26.4 ± 1.2	2.3 ± 0.6	3.1 ± 1.4	7 ± 1
AD summary	82 ± 2.6	25% F	26.8 ± 1.7	13.6 ± 2.3	5.6 ± 0.2	10.5 ± 0.6	11 ± 2

AD, Alzheimer’s disease; Ctl, control; MCI, mild cognitive impairment; MMSE, mini-mental state examination; N/A, not applicable; PMI, *postmortem* interval. Summary data indicate means ± SEM, or percentage.

Table S3. Antibodies used in this study

Antibody target	Source	Cat. No.	Species/clonality	Application	Dilution	Figures
**53BP1**	Novus	NB100-304	Rabbit/Polyclonal	IHC	1:10000	2, S4
**53BP1**	Novus	NB100-304	Rabbit/Polyclonal	IHC	1:1000	S6, S8
**53BP1**	Abcam	ab87097	Rabbit/Polyclonal	IHC	1:1000	S7
**Amyloid-β 82E1**	IBL AMERICA	10323	Mouse/Monoclonal	IHC	1:500	S5
**Biotin**	Abcam	Ab53494	Rabbit/Polyclonal	DI-PLA	1:2000	6, 7d, S9
**Calbindin**	Abcam	AB82812	Mouse/Monoclonal	IHC	1:1000	S5
**GFAP**	Millipore	IF03L	Mouse/Monoclonal	IHC	1:500	1, 2, S4
**γH2AX**	Millipore	MABE205	Rabbit/Monoclonal	IHC	1:100	1, S2b, S4
**γH2AX**	Millipore	MABE205	Rabbit/Monoclonal	ICC	1:1000	4c, 5c
**γH2AX**	Abcam	ab26350	Mouse/Monoclonal	IHC	1:1000	3, 4a, 7b, 7c, S1, S2a, S3, S6, S8
**γH2AX**	Abcam	ab26350	Mouse/Monoclonal	DI-PLA	1:4000	6, 7d, S9
**NeuN**	Millipore	MAB377	Mouse/Monoclonal	IHC	1:2000	1, 2, S2b, S4
**NeuN**	Abcam	ab104224	Mouse/Monoclonal	IHC	1:1000-1:2000	3, 4a, 5c, 6, 7, S1, S2a, S3, S6, S9
**NeuN**	Abcam	ab104224	Mouse/Monoclonal	ICC	1:2000	4c
**Parvalbumin**	Swant	PV27	Rabbit/Polyclonal	IHC	1:3000	3
**Phospho-Tau PHF1**	Dr. Peter Davis	N/A	Mouse/Monoclonal	IHC	1:2000	S5

DI-PLA, DNA damage *in situ* ligation followed by proximity ligation assay; ICC, immunocytochemistry; IHC, immunohistochemistry; N/A, not applicable

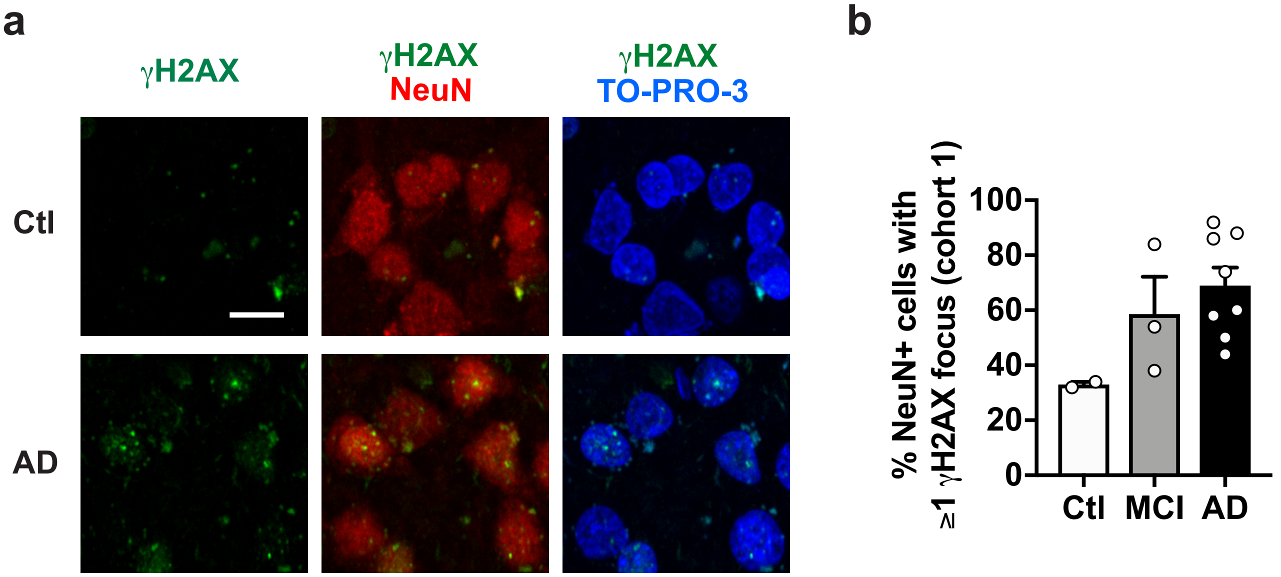

Figure S1 Neuronal γH2AX labeling in frontal cortex of humans with or without MCI or AD. **a** Representative confocal microscopic images of *postmortem* frontal cortex sections from a cognitively unimpaired control (Ctl) and a case with AD of Cohort 1. Samples were immunostained for γH2AX (green) and the neuronal marker NeuN (red). Nuclei were stained with TO-PRO-3 (blue). Scale bar: 10 μm. **b** Proportion (%) of neurons with at least one γH2AX focus in the frontal cortex of cognitively unimpaired controls (Ctl, n = 2) and cases with MCI (n = 3) or AD (n = 8) from Cohort 1. Bars indicate mean ± SEM and dots individual cases.

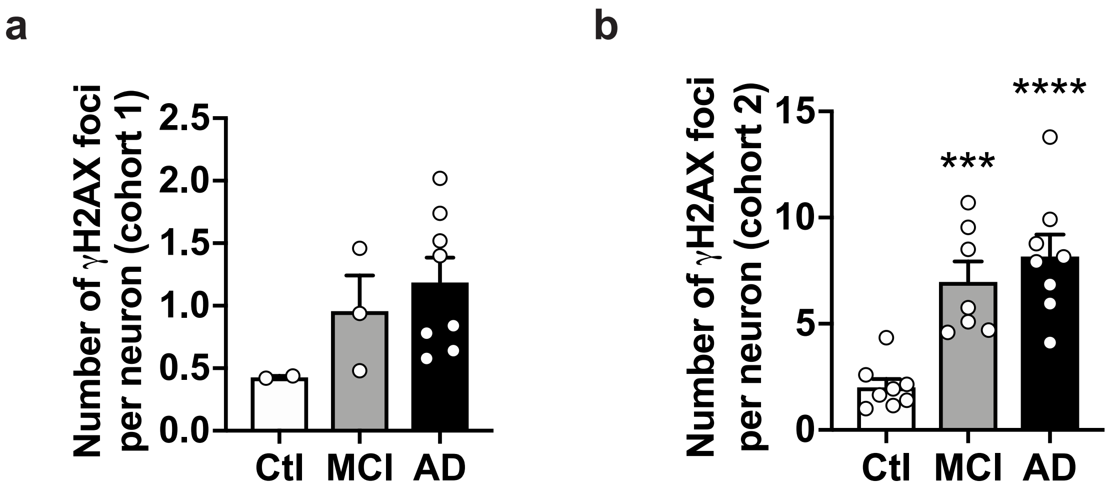

Figure S2 Neuronal γH2AX labeling in frontal cortex of humans with or without MCI or AD. **a** Number of γH2AX foci per neuron in the frontal cortex in cognitively unimpaired controls (Ctl) and cases with MCI or AD of Cohort 1 (n = 2 Ctl, 3 MCI, 8 AD). **b** Number of γH2AX foci per neuron in the frontal cortex in cases of Cohort 2 (n = 8 Ctl, 7 MCI, 8 AD). ***$p < 0.001$, ****$p < 0.0001$ vs. Ctl by one-way ANOVA and Holm-Sidak test. Bars indicate mean ± SEM and dots individual cases.

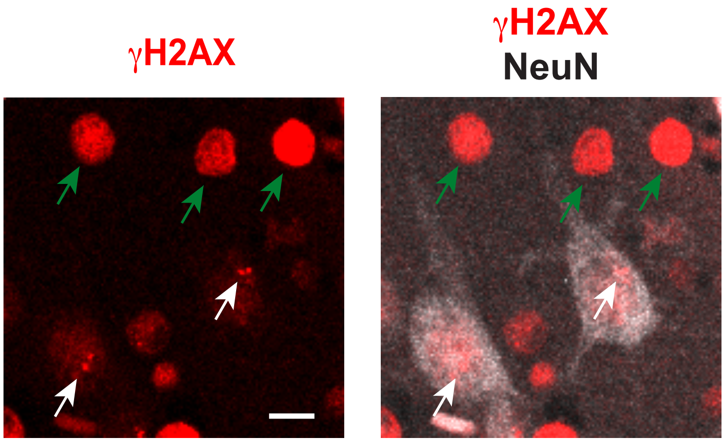

Figure S3 Non-neuronal pan-nuclear H2AX phosphorylation in AD. Representative confocal microscopic images of frontal cortex sections from an AD case of Cohort 1. *Left*: γH2AX alone. *Right*: Merge of γH2AX (red) and NeuN co-labeling (gray). White arrows indicate γH2AX foci suggestive of DSBs in neurons. Green arrows indicate pan-nuclear γH2AX labeling in non-neuronal cells. Scale bar: 10 μm.

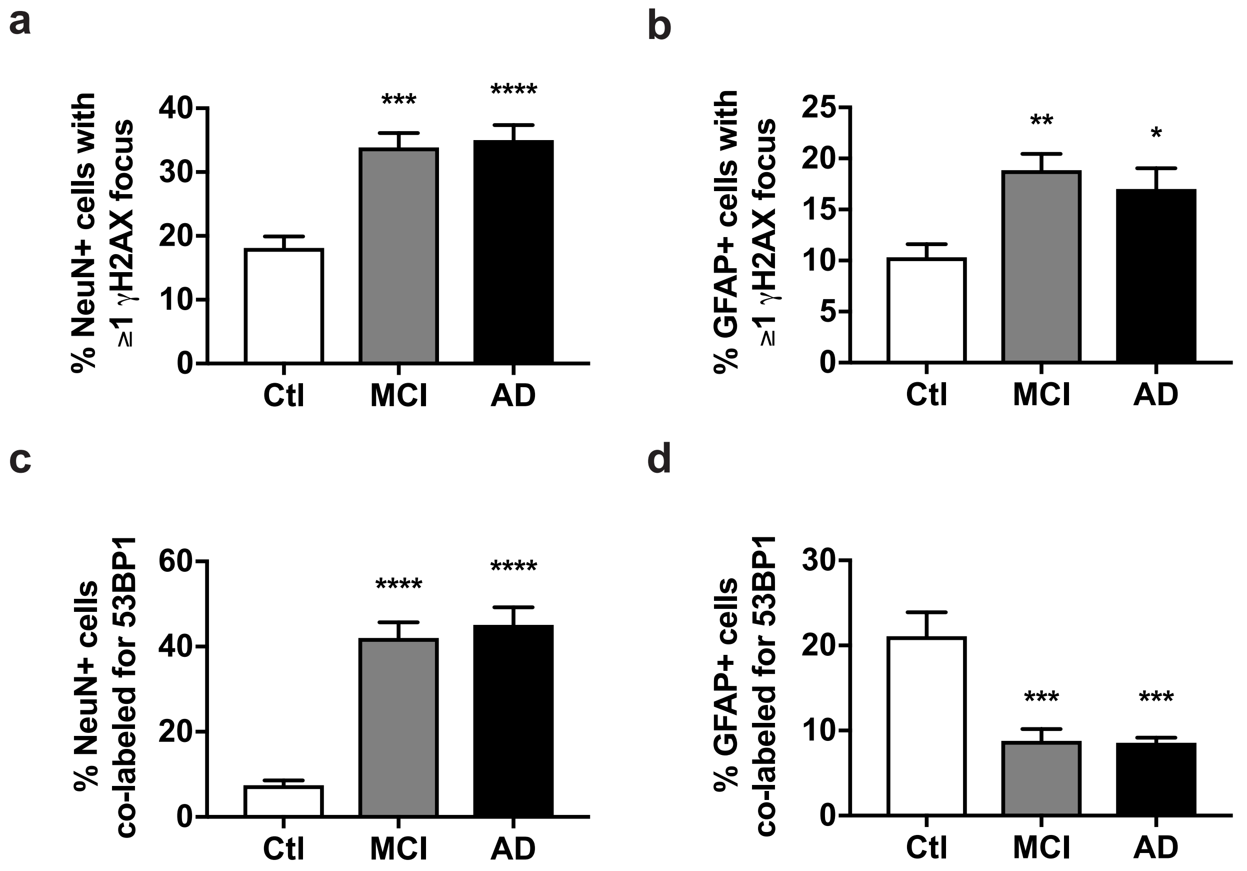

Figure S4 Neuronal and astroglial γH2AX and 53BP1 labeling in the hippocampal CA1 region of cases with MCI or AD. **a, b** Proportion (%) of neurons (**a**) and astrocytes (**b**) with one or more γH2AX foci in cognitively unimpaired controls (Ctl) and cases with MCI or AD. **c, d** Proportion (%) of 53BP1-labeled neurons (**c**) and astrocytes (**d**) in the same cases. n = 8 Ctl, 7 MCI and 8 AD cases from Cohort 2. *p < 0.05, **p < 0.01, ***p < 0.001, ****p < 0.0001 vs. Ctl by one-way ANOVA and Holm-Sidak test. Bars represent means ± SEM.

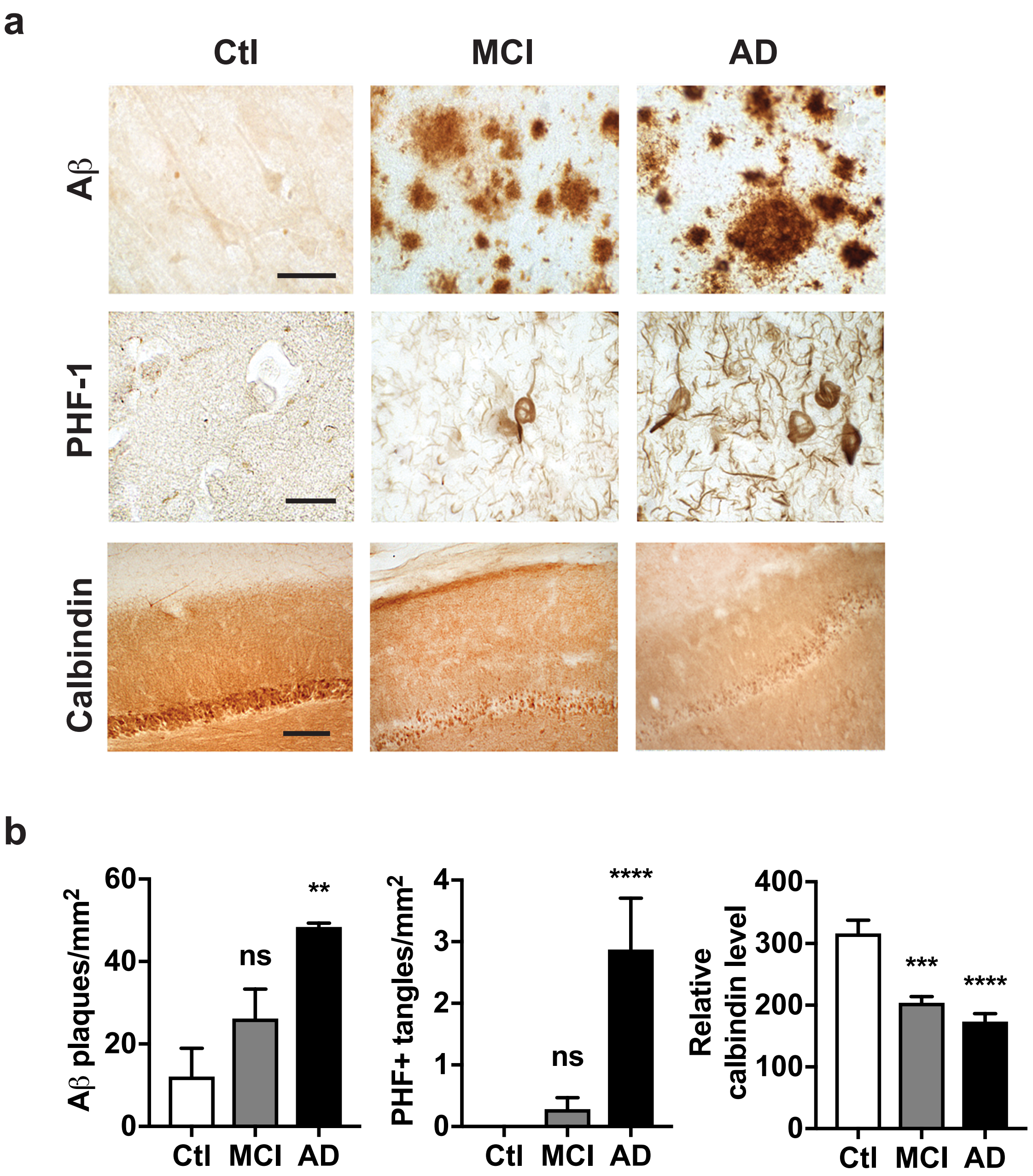

Figure S5 Neuropathological alterations in human Cohort 2. Sections of the frontal cortex were immunoperoxidase-stained for Aβ (4G8; top), phosphorylated tau (PHF-1; middle), or calbindin (bottom). **a** Representative light microscopic images. Scale bars: 20 μm (top and middle), 100 μm (bottom). **b** Quantitations of Aβ deposits, PHF-1-positive inclusions, and relative densities of calbindin immunoreactivity. n = 8 cognitively unimpaired controls (Ctl), 7 MCI and 8 AD cases. $^{**}p < 0.01$, $^{***}p < 0.001$, $^{****}p < 0.0001$ vs. Ctl by Kruskall-Wallis and Holm-Sidak test. ns, not significant. Bars represent means ± SEM.

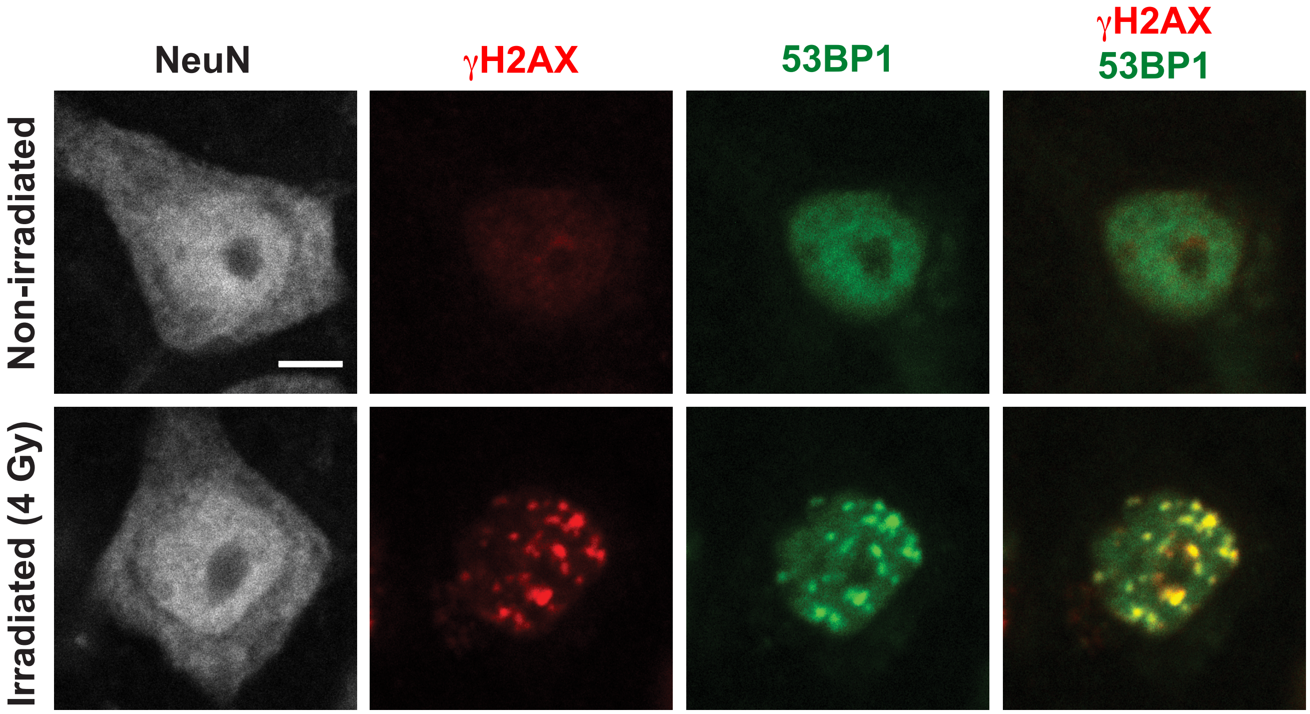

Figure S6 Ionizing radiation causes focal accumulation of γH2AX and 53BP1 in neuronal nuclei of mice. Representative confocal microscopic images of somatosensory cortex sections immunostained for NeuN (gray), γH2AX (red) and 53BP1 (green) from mice that were not irradiated (top) or exposed to whole-body ionizing radiation (4 Gy) 1 h before analysis (bottom). Scale bar: 10 μm.

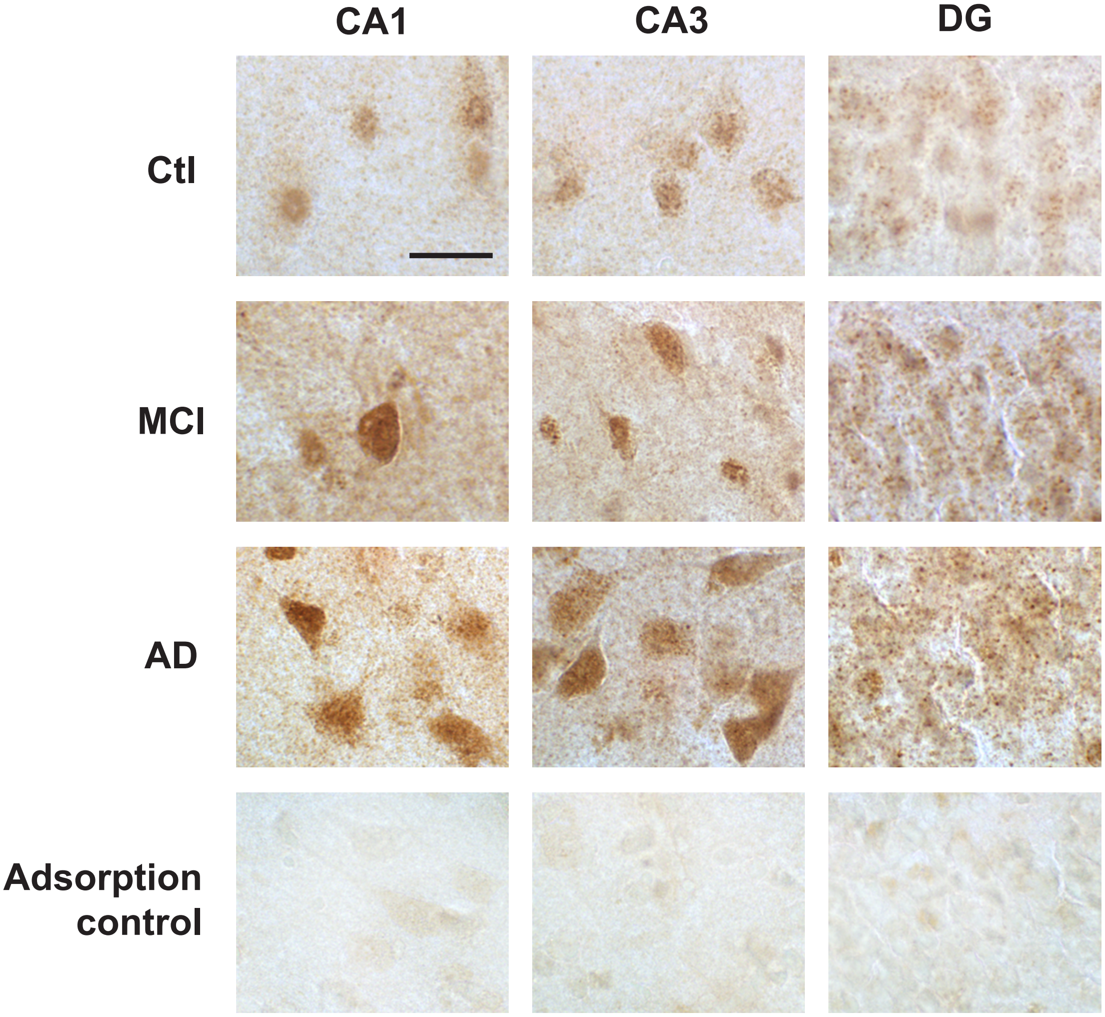

Figure S7 Increased hippocampal 53BP1 staining in humans with MCI or AD. Sections from the indicated cases (Cohort 2) and brain regions were immunoperoxidase-stained for 53BP1 and imaged by light microscopy. Preadsorption of the antibody with a peptide corresponding to the 53BP1 immunogen eliminated most of the staining (bottom). The preadsorption control was performed on tissues from an AD case. Scale bar: 30 μm.

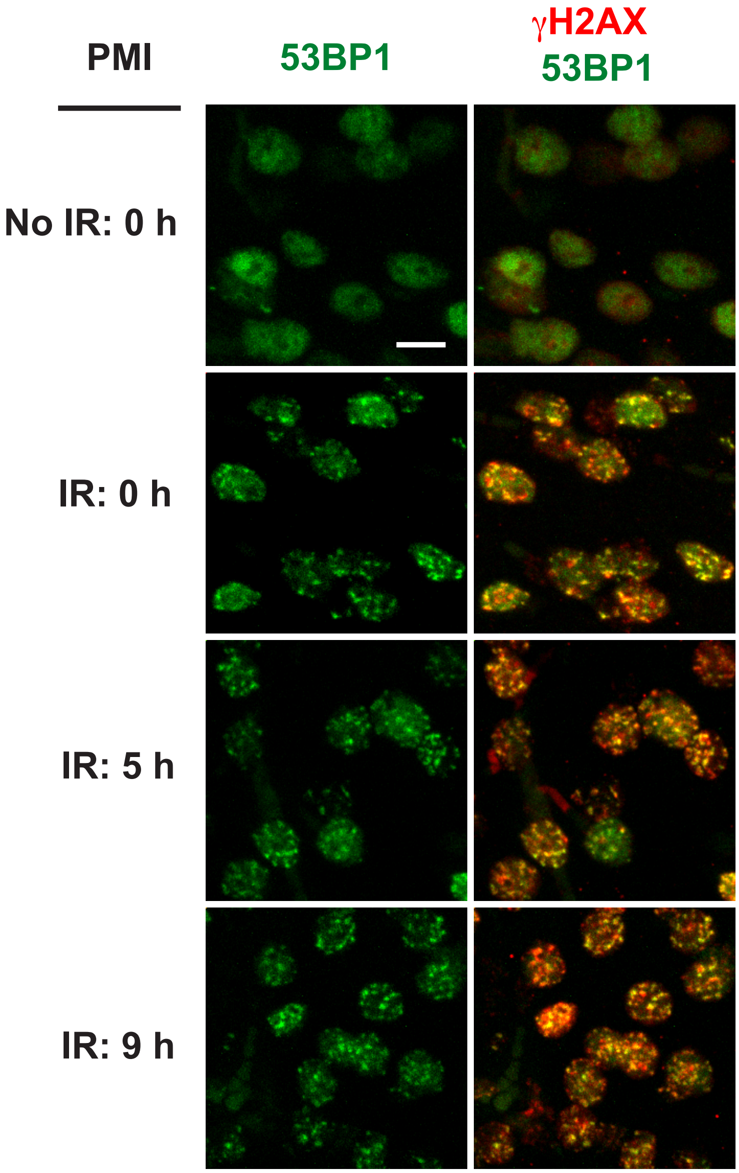

Figure S8 Increasing the *postmortem* interval (PMI) to 9 h does not change 53BP1 or γH2AX labeling of irradiation-induced DSBs in mouse brains. Representative confocal images of somatosensory cortex from 4–6-month-old mice that were not irradiated (top) or exposed to whole-body ionizing radiation (4 Gy) 30 min before sacrifice (other rows). Brains were fixed immediately (PMI: 0 h) or left at room temperature for 2 h and then for 3 or 7 h at 4° C for total PMIs of 5 and 9 h, respectively. All brains were drop-fixed in 10% neutral buffered formalin for 48 h and processed for 53BP1 (green) and γH2AX (red) immunostaining. Scale bar: 10 µm.

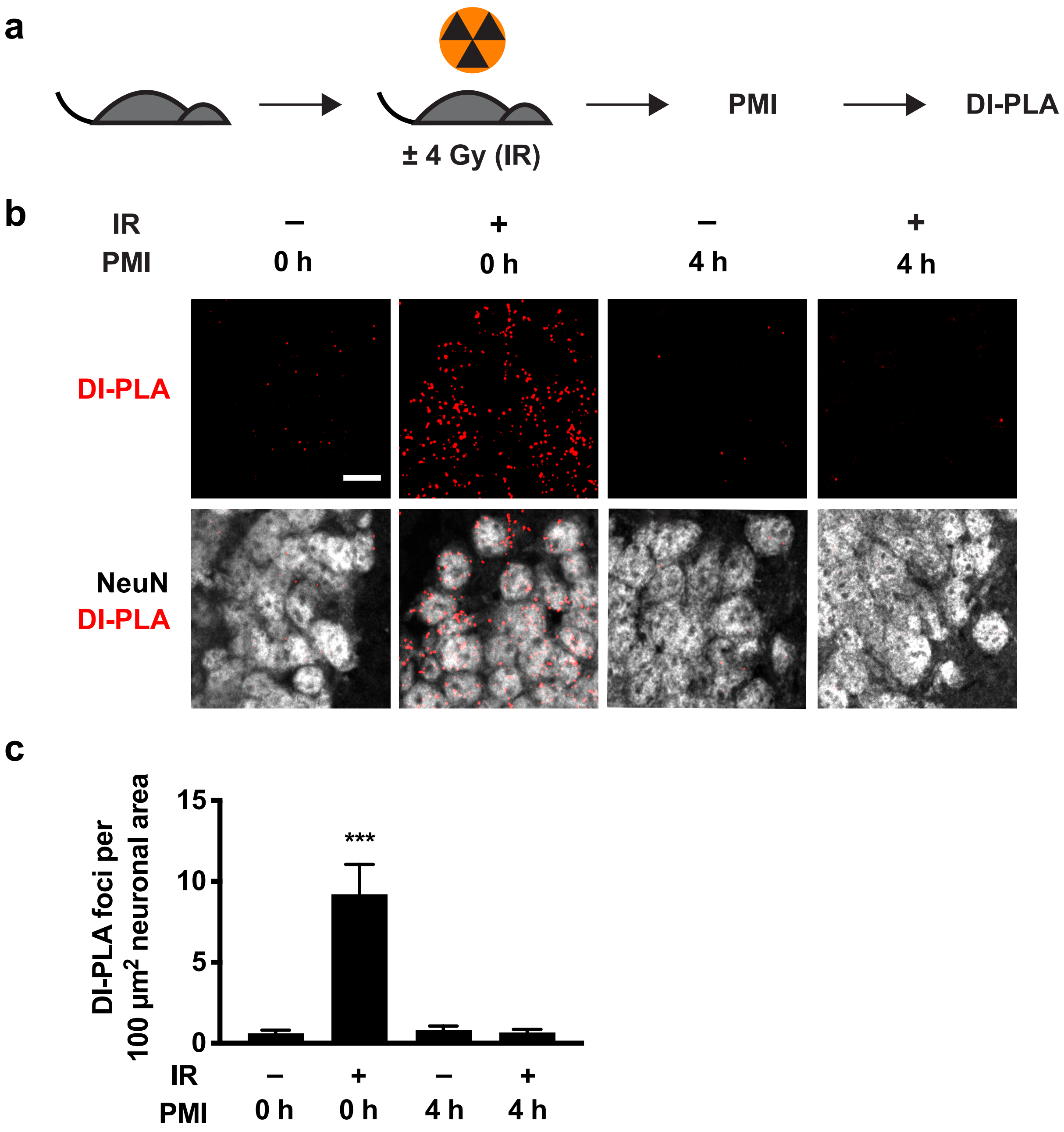

Figure S9 *Postmortem* delays in tissue processing and *postmortem* irradiation do not cause DI-PLA signals in mouse brains. **a** Schematic of experimental design. Mice were or were not exposed to whole-body ionizing radiation (4 Gy) 15 min before they were sacrificed. Brain tissues were processed for DI-PLA immediately after mice were killed or after mice were left for 2 h at room temperature and then for 2 h at 4° C for a total PMI of 4 h. **b** Representative confocal images of dentate gyrus sections. DI-PLA signals (top) and merged view with NeuN co-labeling (bottom) are shown. Scale bar: 10 µm. **c** Number of DI-PLA foci per 100 μm^2 neuronal area in the apex of the dentate gyrus. n = 7–8 mice per group. ***$p < 0.001$ vs leftmost bar by Kruskal-Wallis and Holm-Sidak tests. Bars represent means ± SEM.
